# Heat-responsive *ONSEN* long terminal repeats integrate heat shock factor motifs, DNA methylation and natural sequence variation in *Arabidopsis*

**DOI:** 10.1242/bio.062799

**Published:** 2026-09-11

**Authors:** Baibhav R. Barbaruah, Rahmadani P. Airlangga, Hidetaka Ito

**Affiliations:** ^1^ Division of Life Science, Graduate School of Life Science, Hokkaido University, Sapporo 060-0810, Japan; ^2^ Faculty of Science, Hokkaido University, Sapporo 060-0810, Japan

**Keywords:** *Arabidopsis thaliana*, *ONSEN*, Heat shock factor, Heat shock element, DNA methylation, Retrotransposon

## Abstract

*ONSEN* is a heat-activated Ty1/copia retrotransposon in *Arabidopsis thaliana* controlled by heat shock factors (HSFs) and epigenetic silencing. Heat shock element (HSE)-like sequences in *ONSEN* long terminal repeats (LTRs) contribute to heat responsiveness, but relationships among sequence architecture, basal DNA methylation and natural variation remain unclear. We combined transcription-factor motif prediction, transposable-element comparisons, methylome and RNA sequencing (RNA-seq) data, and *Arabidopsis* genome assemblies. *In silico* disruption of five HSE cores eliminated HSF-family motif compatibility in the selected design and all 5119 exact-guanine–cytosine (GC) alternatives. Across 16 curated Columbia-0 terminal windows, *ONSEN* contained 33–49 non-redundant HSF motif-coordinate placements per 800 bp window and was strongly enriched relative to 1930 non-*ONSEN* transposable elements across score thresholds and continuous metrics. Direct comparison with 779 non-*ONSEN* LTR retrotransposons showed selectively elevated basal CHH methylation (where H = A, C or T) at *ONSEN* termini. Genome-wide RNA-seq analysis revealed broad heat-responsive gene and transposable-element changes, including strong *ONSEN* induction, whereas candidate-window analysis distinguished *ONSEN* from most HSF-rich non-*ONSEN* outliers. *ONSEN*-like variants across eight accessions generally retained HSF-compatible motifs while altering predicted DNA binding with one finger-family motif composition. Together, these findings define *ONSEN* terminal regions as HSF-rich regulatory sequences that retain heat-responsive potential within a methylated chromatin context and identify candidates for functional analysis.

## Teaser

**Summary:** *ONSEN* terminal regions combine non-redundant heat shock factor motif enrichment, elevated basal CHH methylation relative to non-*ONSEN* LTR retrotransposons, heat-associated transcription and motif conservation across natural variants.

## INTRODUCTION

Transposable elements (TEs) occupy a substantial proportion of plant genomes and contribute to genome evolution by generating insertional polymorphisms, altering gene expression and creating genetic diversity. Because uncontrolled transposition threatens genome integrity, most TEs are epigenetically silenced through DNA methylation and small RNA-mediated pathways (Matzke and Mosher, 2014). Nevertheless, some TEs escape this repression under environmental stress, allowing stress-responsive regulatory pathways to influence their transcriptional activity (Lisch, 2013; Baduel et al., 2021).

Heat stress provides a well-characterised example of this interaction. In *Arabidopsis thaliana*, prolonged heat stress induces chromatin decondensation and activates a limited number of stress-responsive retrotransposons without extensive genome-wide loss of DNA methylation (Pecinka et al., 2010). Consequently, TE activation under heat stress is thought to depend on both stress-responsive transcription factors and the surrounding epigenetic landscape.

Among these elements, *ONSEN* is one of the best-characterised heat-responsive Ty1/copia retrotransposons in *Arabidopsis*. *ONSEN* transcription is rapidly induced by heat stress, whereas transgenerational transposition occurs most efficiently in mutants defective in RNA-directed DNA methylation or small RNA biogenesis (Ito et al., 2011; Matsunaga et al., 2012, 2015; Hayashi et al., 2020). *ONSEN* therefore represents an excellent model for understanding how environmental stress interacts with transcriptional regulation and epigenetic silencing.

A central feature of *ONSEN* regulation is the presence of heat shock element (HSE)-like sequences within its long terminal repeats (LTRs). These motifs are recognised by heat shock factors (HSFs), which coordinate transcriptional responses to elevated temperature. Previous studies demonstrated that HSFA2 contributes to *ONSEN* activation and that disruption of HSEs reduces heat responsiveness (Schramm et al., 2006; Charng et al., 2007; Cavrak et al., 2014; Matsunaga et al., 2015). However, HSEs are embedded within larger regulatory regions that may contain multiple overlapping cis-elements. Consequently, sequence changes designed to disrupt HSF recognition may also alter overlapping predicted motifs, and their effects must be interpreted in relation to the specific substitutions introduced.

Epigenetic regulation provides an additional layer of control. *ONSEN* remains heavily methylated under normal conditions despite possessing heat-responsive cis-elements, and natural accessions differ substantially in *ONSEN* copy number, genomic distribution, DNA methylation and heat-induced expression (Masuda et al., 2017; Nozawa et al., 2022; Xu et al., 2024). These observations indicate that sequence architecture, chromatin state and genomic context are all associated with *ONSEN* activity, but their relationships have not been compared systematically.

Here, we combined motif analyses, genome-wide TE comparisons, public DNA methylation data, RNA sequencing (RNA-seq) data generated in this study, and chromosome-level assemblies to define *ONSEN* terminal-region architecture. This design used non-redundant motif-coordinate placements to quantify HSF compatibility, tested predicted HSF loss across constrained alternative sequences, directly compared *ONSEN* and non-*ONSEN* LTR-retrotransposon methylation, and related terminal architecture to global and candidate-window heat-responsive RNA-seq signals and accession-level sequence variation.

## RESULTS

### The designed HSE-disrupted sequence shows robust loss of HSF-family motif compatibility

**Fig. 1.**
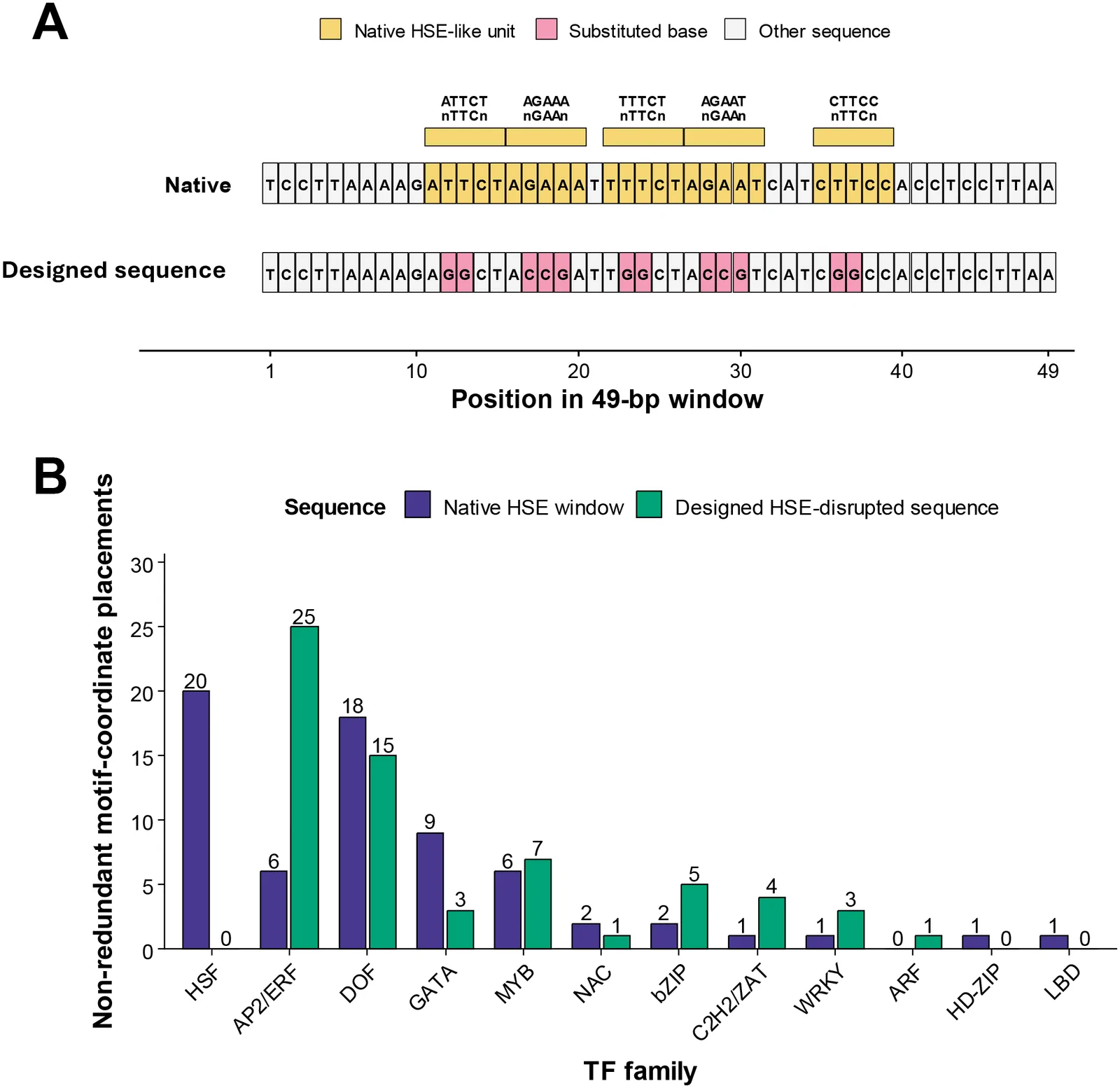
**Native and *in silico*-designed HSE-disrupted *ONSEN* sequences and their predicted transcription-factor motif composition.** (A) Schematic representation of the native 49 bp *ONSEN* HSE-containing sequence and the corresponding *in silico*-designed HSE-disrupted sequence. The native sequence contains five HSE-like units (ATTCT, AGAAA, TTTCT, AGAAT and CTTCC). The designed sequence retains the same length and all non-targeted positions but carries 12 substitutions that disrupt the TTC or GAA cores of these units; nucleotide composition was not preserved (GC content, 30.61% in the native sequence and 51.02% in the designed sequence). (B) Family-level distribution of non-redundant motif-coordinate placements in the two sequences. Duplicate PWM-model/strand predictions with identical 1-based start–end coordinates were collapsed within each TF family. Counts from different families should not be summed because families can overlap at the same coordinates. High-confidence predictions were defined using a relative PWM score threshold of ≥0.85. This single designed sequence is an illustrative perturbation and does not represent a general model of all HSE-disrupting mutations. Motif predictions indicate sequence compatibility and do not represent direct transcription-factor binding *in vivo*.

To examine how a defined HSE-disrupting design affects local predicted motif compatibility, we analysed a native 49 bp *ONSEN* LTR sequence containing five clustered HSE-like units together with an *in silico*-designed sequence of identical length carrying 12 targeted substitutions (Fig. 1A). The substitutions disrupted the TTC or GAA cores of all five units (ATTCT, AGAAA, TTTCT, AGAAT and CTTCC), and no HSE-like units were detected in the designed sequence using the same identification criteria. The native-versus-designed comparison was therefore treated as a case-specific illustration, and its robustness was evaluated using constrained alternative sequence designs.

Motif-database predictions were summarised at two non-redundant levels: distinct position-weight matrix (PWM)-model identities and unique 1-based motif coordinates after duplicate PWM-model/strand predictions with identical start–end coordinates were collapsed. At the PWM-model level, 60 models were classified as lost, 50 as gained, 44 as retained, three as strengthened, two as weakened and three as shifted in position (Table S1). At the transcription-factor-family level, the native sequence contained 20 non-redundant HSF motif-coordinate placements, whereas the designed HSE-disrupted sequence contained none (Fig. 1B; Table S1). These summaries avoid interpreting overlapping predictions from related PWM models as independent sites.

**Fig. 2.**
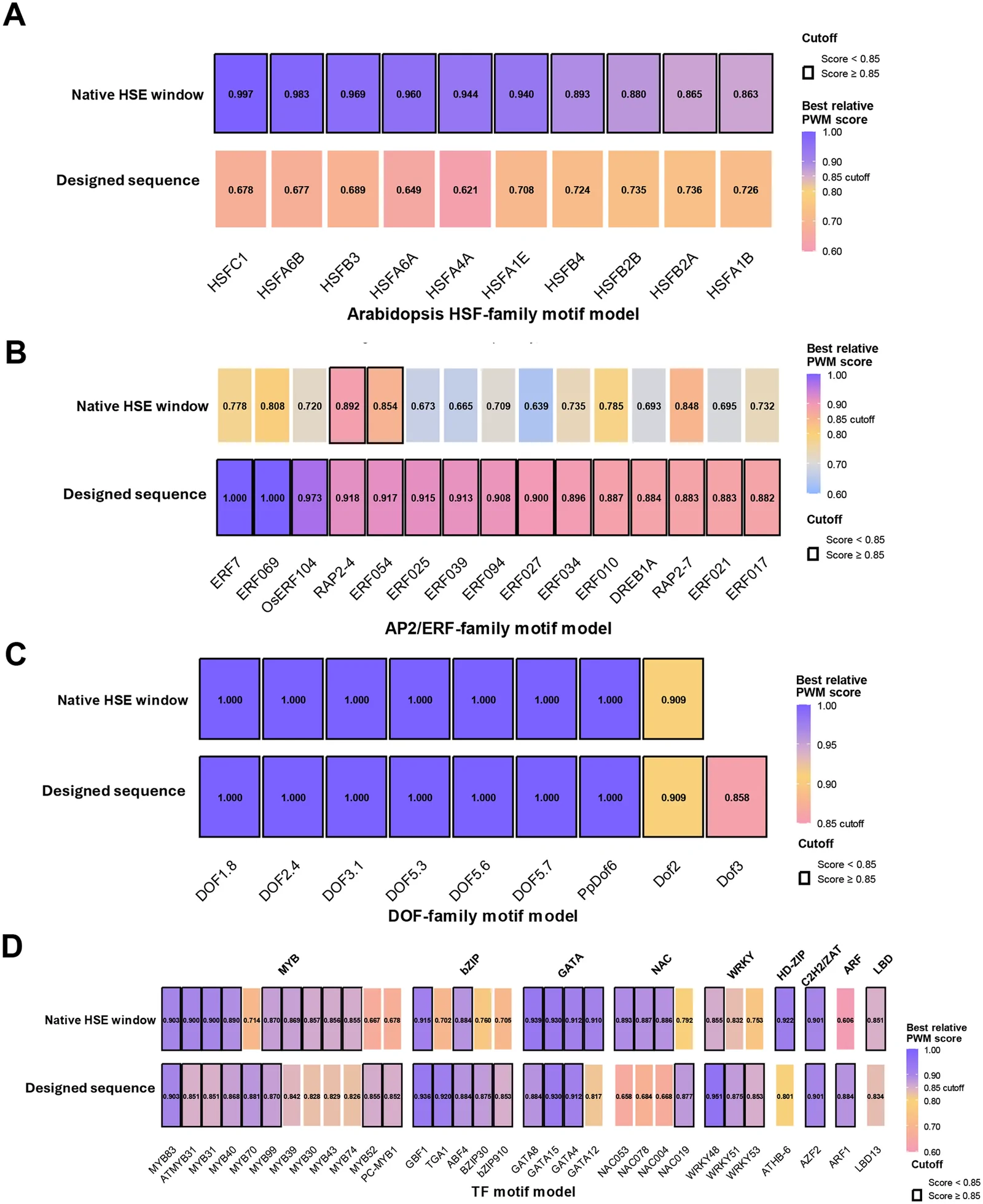
**Predicted transcription-factor PWM-score profiles of the native and *in silico*-designed HSE-disrupted *ONSEN* sequences.** The row labelled ‘Designed sequence’ in each panel represents the same *in silico*-designed HSE-disrupted sequence shown in Fig. 1. (A) Best relative PWM scores for ten *Arabidopsis* HSF-family models. (B) AP2/ERF-family models with high scores in the designed sequence. (C) DOF-family models identified in the two sequences. (D) Representative models from additional transcription-factor families. Tile colours indicate the highest relative PWM score for each model, and numbers show the corresponding score. Black borders denote high-confidence predictions (a relative PWM score of ≥0.85). Differences outside the HSF family are treated as case-specific collateral effects of this substitution scheme and not as general consequences of HSE disruption. Predictions indicate sequence compatibility and not transcription-factor binding *in vivo*.

The native sequence produced above-threshold scores for ten *Arabidopsis* HSF PWM models, namely, HSFA1B, HSFA1E, HSFA4A, HSFA6A, HSFA6B, HSFB2A, HSFB2B, HSFB3, HSFB4 and HSFC1, whereas all ten scores fell below the threshold in the designed sequence (Fig. 2A; Table S2). Other transcription-factor families also showed coordinate and score changes after introduction of the selected substitutions, including AP2/ERF and DOF models (Figs 1B and 2B–D), but these collateral changes were treated only as properties of this particular sequence design and not as general consequences of HSE disruption.

To evaluate whether predicted HSF loss depended on the selected design, we generated 5000 unique constrained random mutants in which exactly 12 of the 15 HSE-core positions were substituted, all five native HSE-like cores were disrupted, and no canonical nTTCn or nGAAn unit remained anywhere in the 49 bp sequence. GC content was not constrained. The selected design contained zero non-redundant HSF motif-coordinate placements at the primary 0.85 threshold, and the constrained-mutant distribution was also concentrated at zero, with a maximum of one HSF prediction per sequence (Table S3). Because no comparator contained more than one prediction, exact-coordinate deduplication did not alter this distribution.

We then exhaustively enumerated the complete valid exact-GC design space under the same substitution and HSE-disruption constraints. This yielded 5120 valid sequences: the original designed sequence and 5119 alternatives with the same 51.02% GC content. The selected design and all 5119 alternatives contained zero non-redundant HSF motif-coordinate placements at the 0.85 threshold (Table S3). Thus, predicted HSF-family motif loss was robust within the imposed design space, although this computational result does not establish transcription-factor occupancy or regulatory function *in vivo*.

### *ONSEN* terminal candidate windows are enriched for HSF-family motifs across the *Arabidopsis* genome

**Fig. 3.**
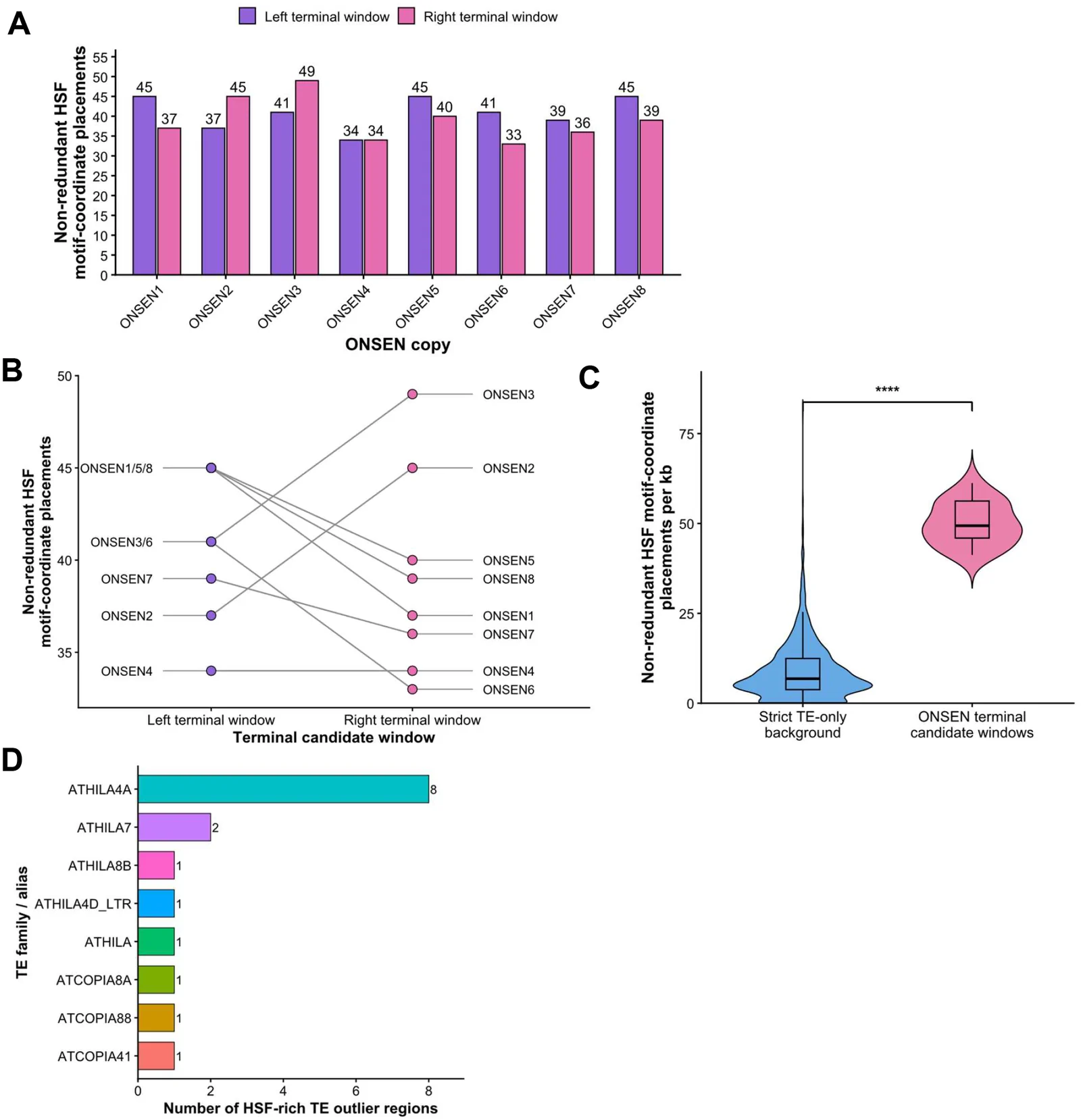
**Non-redundant HSF-family motif-coordinate placement density across Col-0 *ONSEN* terminal candidate windows and background TEs.** (A) Number of non-redundant HSF motif-coordinate placements within fixed 800 bp windows from both terminal ends of eight historically curated Col-0 *ONSEN* loci. Duplicate PWM-model/strand predictions with identical start–end coordinates were collapsed. These windows are operational candidate regions rather than experimentally validated exact LTR boundaries or updated copy-integrity calls. (B) Comparison between paired left and right terminal windows from each curated locus. Connected points represent paired windows from the same locus. (C) Distribution of non-redundant HSF placement density in 16 *ONSEN* terminal candidate windows and 1930 strict non-*ONSEN* TE regions at the primary 0.85 cut-off (two-sided Wilcoxon *P*=6.97×10^−12^; BH-adjusted *P*=7.09×10^−12^; Cliff's δ=0.993; ****adjusted *P*<0.0001). (D) TE-family assignments of the HSF-rich non-*ONSEN* outlier regions after *ATCOPIA78*/*ONSEN* annotation harmonisation. Strict overlap-merged HSF regions are shown in Fig. S1 and Table S5; extended threshold and continuous-score comparisons are shown in Fig. S2 and Table S7.

Columbia-0 (Col-0) has historically been described as containing eight full-length *ONSEN* copies (Cavrak et al., 2014; Nozawa et al., 2021). We therefore analysed both terminal ends of this established eight-locus set, yielding 16 fixed 800 bp terminal candidate windows (Fig. 3A,B). These operational windows were retained consistently across the motif, methylation and RNA-seq analyses; they should not be interpreted as updated copy-integrity calls or experimentally validated exact LTR boundaries. Every window contained 33–49 non-redundant HSF motif-coordinate placements (41.25–61.25 placements kb^−1^), and the windows contained nine or ten distinct *Arabidopsis* HSF PWM models (Table S4).

The principal non-redundant metric collapsed duplicate PWM-model/strand predictions only when their 1-based start and end coordinates were identical. As a stringent sensitivity analysis, we also merged HSF intervals transitively whenever they overlapped by at least one nucleotide. In the native 49 bp sequence, 20 exact-coordinate placements collapsed to one strict overlap-merged HSF region, whereas the designed sequence contained none. Across the 16 Col-0 *ONSEN* terminal windows, 33–49 exact-coordinate placements corresponded to 7–10 strict overlap-merged regions per window, with a median of 8 (Fig. S1; Table S5). Both metrics describe predicted sequence compatibility rather than independent binding sites or HSF occupancy.

To determine whether HSF-rich predicted architecture is characteristic of *ONSEN*, non-redundant motif-coordinate placement densities were compared with a stringent background consisting exclusively of annotated TE regions. At the primary relative PWM-score cut-off of 0.85, the median HSF placement density was 49.375 placements kb^−1^ in *ONSEN* terminal candidate windows and 6.812 placements kb^−1^ in 1930 strict non-*ONSEN* TE regions [two-sided Wilcoxon rank-sum *P*=6.97×10^−12^; Benjamini–Hochberg (BH)-adjusted *P*=7.09×10^−12^; Cliff's δ=0.993; Fig. 3C; Table S6].

To assess whether this enrichment depended on the selected score cut-off, we repeated the comparison at relative PWM-score thresholds of 0.80, 0.85, 0.90 and 0.95. Median *ONSEN* placement densities were 104.375, 49.375, 24.375 and 5.000 placements kb^−1^, respectively, compared with 27.730, 6.812, 0.939 and zero placements kb^−1^ in the strict non-*ONSEN* TE background. Effect sizes remained extremely large across all four thresholds (Cliff's δ=0.974–0.994; Fig. S2A; Table S7).

Threshold-independent regional summaries produced the same conclusion. The maximum HSF relative PWM score, the mean of the five highest HSF scores and the median of the ten per-model maximum scores were all significantly higher in *ONSEN* terminal candidate windows than in the strict non-*ONSEN* TE background (BH-adjusted two-sided Wilcoxon *P*≤2.81×10^−8^; Fig. S2B,C; Table S7). Thus, the HSF-rich predicted architecture of *ONSEN* terminal candidate windows is not an artefact of redundant PWM predictions or a single binary score threshold.

A limited number of non-*ONSEN* TEs also displayed relatively high non-redundant HSF placement densities. After harmonising *ATCOPIA78* annotations with the *ONSEN* family, most remaining outliers belonged to the *ATHILA4A* family rather than being broadly distributed among unrelated TEs (Fig. 3D). Thus, *ONSEN* terminal candidate windows rank among the most HSF-enriched TE regions in *Arabidopsis*.

### *ONSEN* terminal candidate regions show selectively elevated basal CHH methylation relative to non-*ONSEN* LTR retrotransposons

**Fig. 4.**
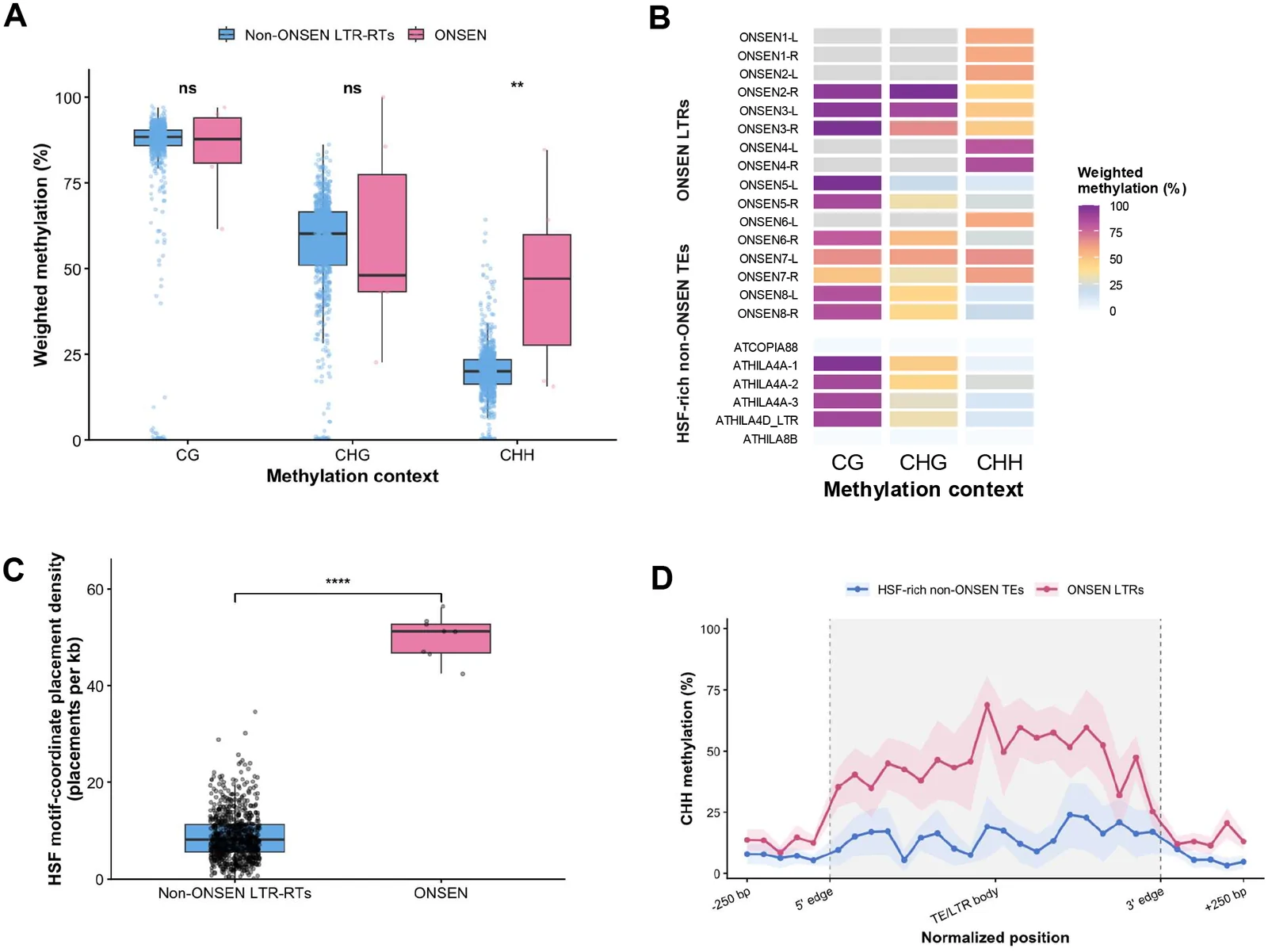
**DNA methylation profiles of *ONSEN* terminal candidate regions and non-*ONSEN* LTR retrotransposons.** (A) Principal element/copy-level comparison of weighted CG, CHG and CHH methylation using public whole-genome bisulphite-sequencing data from unstressed Col-0 leaves (GEO GSE43857; GSM1085222). Left and right terminal-region counts were aggregated per element/copy. Context-specific sample sizes were six, six and eight covered *ONSEN* copies and 779 non-*ONSEN* LTR-retrotransposon elements for CG, CHG and CHH, respectively. Two-sided Wilcoxon rank-sum tests were BH corrected across contexts (ns, adjusted *P*≥0.05; **adjusted *P*<0.01). (B) Locus-level methylation heatmap for *ONSEN* terminal windows and the six members of the fixed eight-region HSF-rich non-*ONSEN* TE subset with complete three-context methylation rows; the full subset and display status are listed in Table S8D. (C) Comparison of non-redundant HSF motif-coordinate placement density between eight *ONSEN* copies and 794 annotation-defined non-*ONSEN* LTR-retrotransposon elements (****P<0.0001). This panel compares motif-density distributions and does not plot methylation. (D) Aggregate CHH methylation profiles across scaled regions and flanks; lines show means, and shading shows s.e.m. These analyses describe basal methylation in unstressed Col-0 leaves and do not measure heat-induced change.

The enrichment of HSF-family motifs across *ONSEN* terminal regions prompted us to examine whether these potentially heat-responsive regions retain the epigenetic characteristics typical of LTR retrotransposons. We integrated public whole-genome bisulphite-sequencing data from unstressed Col-0 leaves with annotation-defined non-*ONSEN* LTR retrotransposons. The principal comparison aggregated terminal-region methylation at the element/copy level to avoid treating paired terminal windows from the same element as independent observations, yielding 779 non-*ONSEN* LTR-retrotransposon elements and six to eight covered *ONSEN* copies, depending on methylation context (Fig. 4A; Table S8A).

Basal CG and CHG methylation (where H = A, C or T) did not differ significantly between *ONSEN* copies and non-*ONSEN* LTR retrotransposons (median 87.76% versus 88.43% for CG and 48.11% versus 60.28% for CHG; BH-adjusted Wilcoxon *P*=0.849 for both contexts; Fig. 4A; Table S8A). In contrast, CHH methylation was higher at *ONSEN* terminal regions than in the direct LTR-retrotransposon comparator (median 47.09% versus 20.06%; BH-adjusted *P*=0.00864; Cliff's δ=0.612). A window-level sensitivity analysis and the broader ordinary-TE control produced the same selective CHH pattern (Table S8B,C).

Locus-level analyses separated three related displays. A heatmap showed basal CG, CHG and CHH methylation across *ONSEN* terminal windows and the six members of the fixed eight-region HSF-rich non-*ONSEN* TE subset with complete three-context methylation rows (Fig. 4B; Table S8D). *ONSEN* copies also had greater non-redundant HSF motif-coordinate placement density than the annotation-defined non-*ONSEN* LTR-retrotransposon comparator (Fig. 4C), and aggregate profiles showed higher basal CHH methylation across *ONSEN* termini (Fig. 4D). Because the methylome was generated from unstressed Col-0 leaves, these data describe only the pre-existing methylation context of *ONSEN* and do not measure methylation dynamics during heat stress.

### Heat stress induces genome-wide gene and TE expression changes and increases *ONSEN*-associated RNA-seq signal

To determine whether the HSF-rich sequence architecture identified above is associated with transcriptional activation during heat stress, we analysed six Col-0 RNA-seq libraries generated in this study: three non-stressed controls and three samples exposed to 37°C for 24 h.

**Fig. 5.**
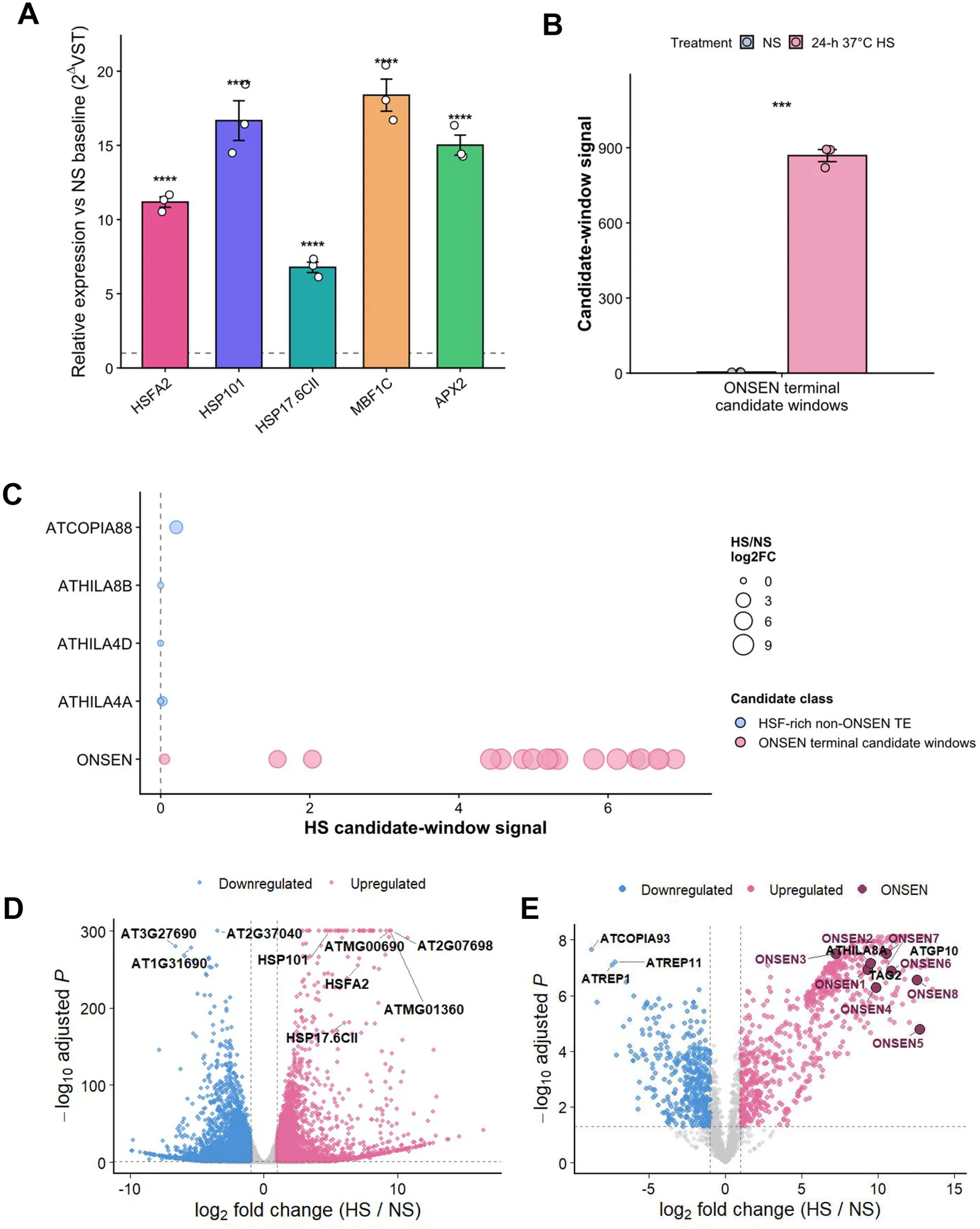
**Genome-wide heat-stress differential expression and RNA-seq signal over *ONSEN* candidate regions after 24 h at 37°C.** The experiment contained *n*=3 biological replicates per condition, and all six libraries were analysed. (A) Relative expression of representative heat-responsive genes. Values were calculated as 2^(VST_sample−mean VST_NS)^; the dashed line at 1 indicates the mean non-stressed baseline. Bars show mean±s.e.m., and points show heat-stressed biological replicates. Asterisks indicate DESeq2 BH-adjusted *P*-values versus non-stressed controls. (B) Fractional CPM summed across the 16 *ONSEN* terminal candidate windows for each biological replicate; bars show mean±s.e.m. (two-sided Welch's *t*-test, *P*=7.57×10^−4^; ***). (C) Heat-stress signal for the 16 curated *ONSEN* terminal windows, two additional *ATCOPIA78* candidate regions and the fixed eight-region HSF-rich non-*ONSEN* TE subset. The horizontal coordinate is log2(mean HS CPM+1); point size represents max{0, log2[(mean HS CPM+0.05)/(mean NS CPM+0.05)]}. (D,E) Volcano plots for all genes with non-missing BH-adjusted *P*-values after DESeq2 independent filtering (D) and all statistically tested TEs after expression filtering (E). Vertical dashed lines mark log2 fold changes of −1 and 1, and the horizontal dashed line marks BH-adjusted *P*=0.05. Upregulated and downregulated calls required adjusted *P*<0.05 and |log2 fold change|≥1. The eight canonical *ONSEN* loci are highlighted and labelled in E. Complete global results are provided in Table S9, and candidate-window values are provided in Table S10.

Gene-level differential expression confirmed a robust heat-stress response. Canonical heat-responsive genes, including *HSFA2*, *HSP101*, *HSP17.6CII*, *MBF1C* and *APX2*, were strongly induced following heat treatment (Fig. 5A). Across the complete gene annotation, DESeq2 returned model estimates for 25,912 of 32,833 genes and raw *P*-values for 25,911; 23,922 genes had non-missing BH-adjusted *P*-values after independent filtering. At BH-adjusted *P*<0.05 and |log2 fold change|≥1, 3912 genes were significantly upregulated, and 4185 were significantly downregulated (Fig. 5D; Table S9).

The global TE analysis retained all 31,189 annotated loci and statistically tested 2101 after expression filtering. Of these, 692 were significantly upregulated, and 397 were significantly downregulated using the same adjusted-*P* and fold-change criteria (Fig. 5E; Table S9). All eight canonical *ONSEN* loci were classified as upregulated, with model log2 fold changes of 7.21–12.71 and BH-adjusted *P*≤1.60×10^−5^. Because *ONSEN* sequences are repetitive, these locus-level model outputs were interpreted together with, but not as a replacement for, the multimapping-aware candidate-window analysis.

RNA-seq signal across the curated *ONSEN* terminal candidate windows increased markedly after heat treatment. Mean fractional counts per million (CPM) per window rose from 0.259 under control conditions to 54.10 after heat stress; Fig. 5B shows the corresponding signal summed across the 16 windows for each biological replicate (Welch's *t*-test, *P*=7.57×10^−4^; Table S10). Fig. 5C displays the 16 curated *ONSEN* terminal windows together with two additional *ATCOPIA78* candidate regions and the fixed eight-region HSF-rich non-*ONSEN* TE subset. Most fixed non-*ONSEN* regions showed little or no comparable increase. These analyses distinguish *ONSEN*-associated heat-responsive signal from most other HSF-rich TE candidates, but they do not prove copy-specific expression or causal HSF occupancy.

### Detected natural *ONSEN*-like HSE-seed variants generally retain HSF-compatible motifs while altering DOF-family predictions

We next examined chromosome-level assemblies of An-1, C24, Cvi-0, Eri-1, Kyo, Ler-1 and Sha together with Col-0 using the Col-0 49 bp *ONSEN* HSE seed. *ONSEN*-like candidate windows were detected in all examined assemblies (Figs S3 and S4; Table S11). Main-chromosome candidate abundance ranged from 7 in An-1 to 41 in Sha (Col-0, 19; C24, 11; Cvi-0, 9; Eri-1, 16; Kyo, 17; Ler-1, 15), and paired HSE/LTR structural proxies ranged from 2 in An-1 to 16 in Sha. These values represent seed-detected candidate windows or paired structural proxies, not definitive *ONSEN* copy numbers.

**Fig. 6.**
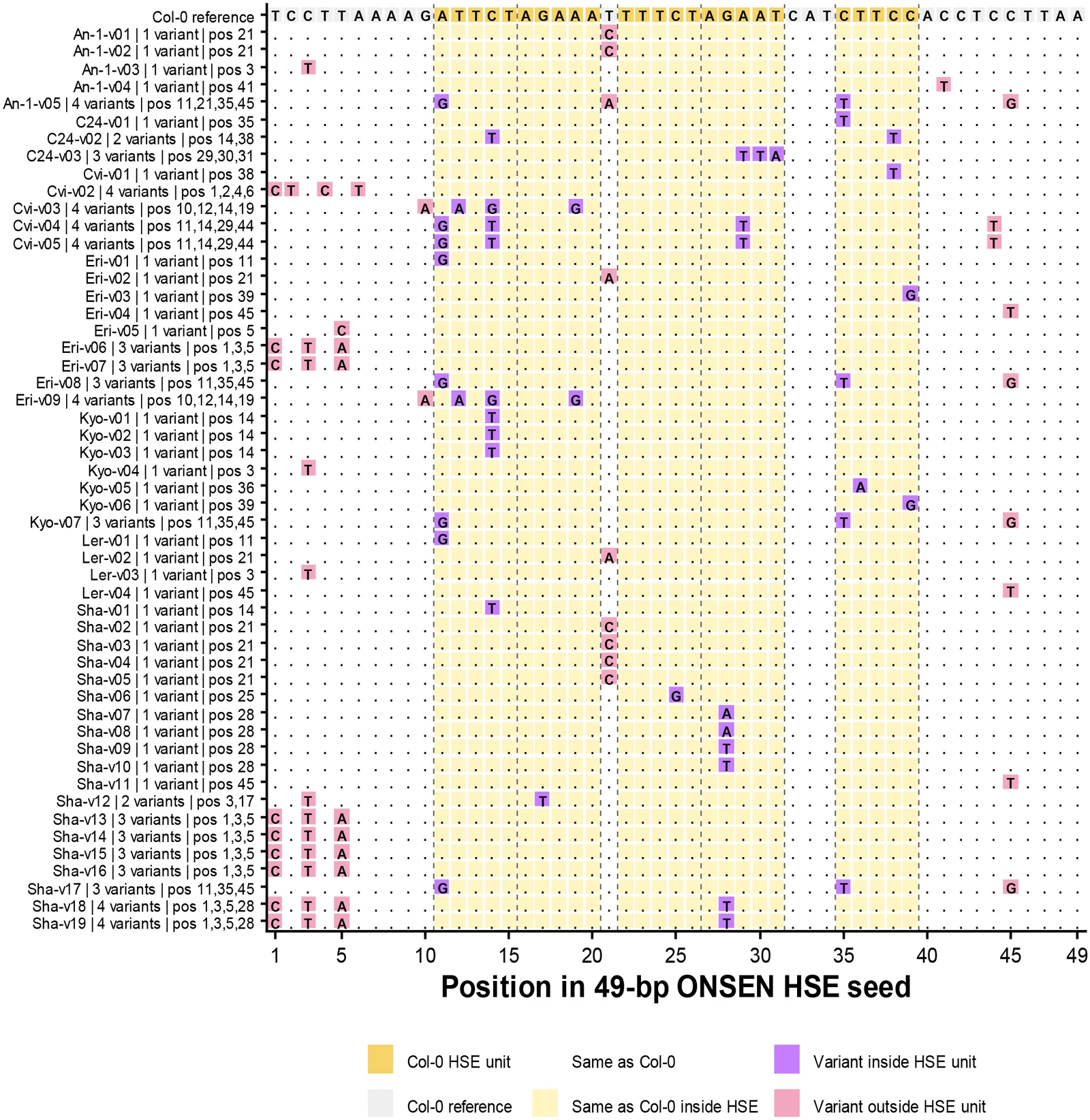
**Sequence variation within *ONSEN* HSE-containing regions across natural *Arabidopsis* accessions.** A representative 49 bp *ONSEN* HSE-containing sequence from the Col-0 reference genome is shown together with non-redundant variant sequences identified in natural *A. thaliana* accessions. Dots indicate nucleotides identical to the Col-0 reference, whereas letters denote sequence differences. Positions of the canonical HSE-like units identified in the Col-0 sequence are highlighted. Sequence variants are shown relative to the selected Col-0 reference seed, not relative to all Col-0 *ONSEN* copies. Accession-level summaries are provided in Table S12.

**Fig. 7.**
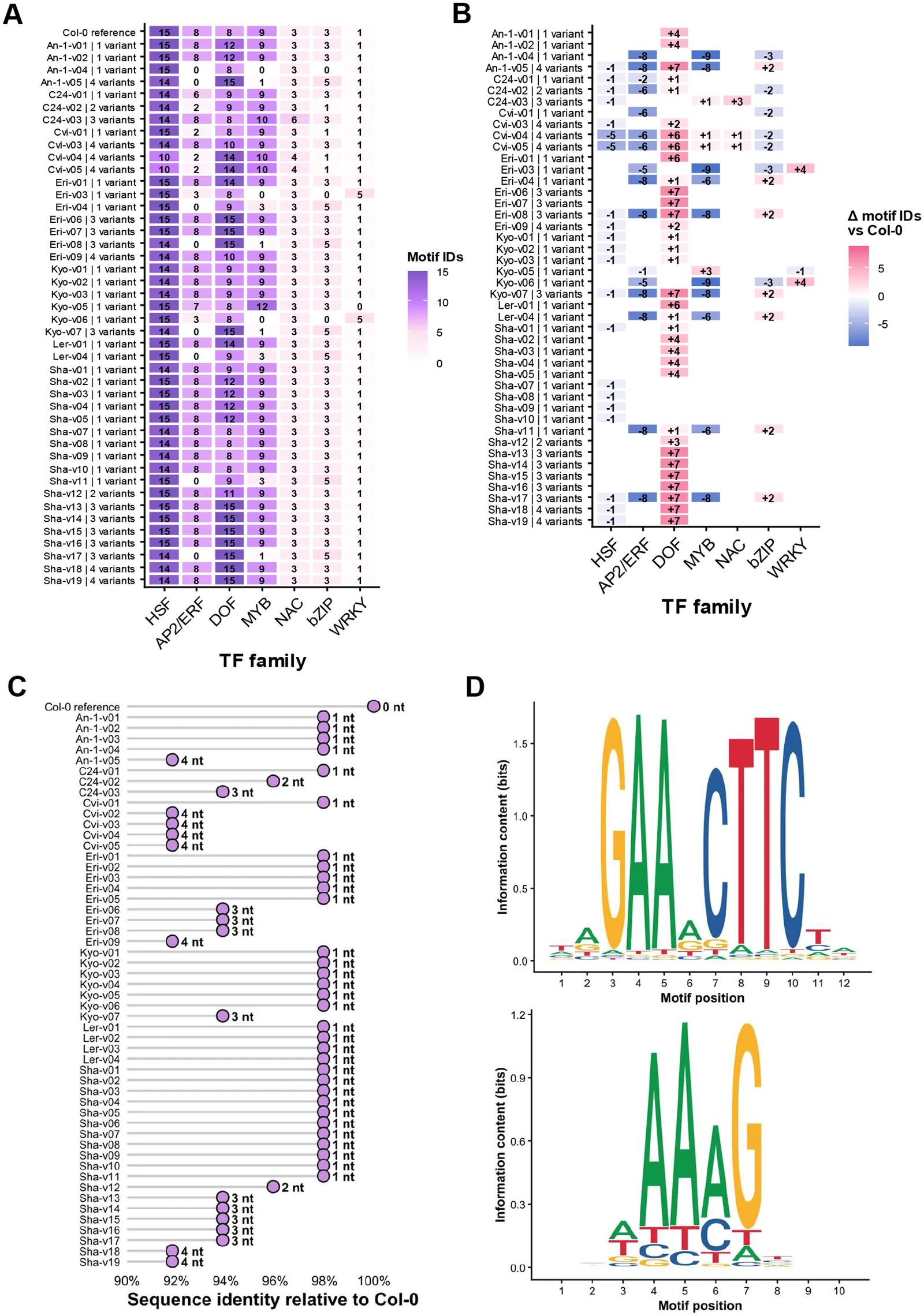
**Sequence identity and predicted transcription-factor-family PWM-model composition of detected natural *ONSEN*-like HSE-seed variants.** (A) Numbers of distinct above-threshold JASPAR PWM-model identities per TF family in the Col-0 reference and detected 49 bp variants. These values are model-identity summaries and not independent motif-coordinate placements or binding sites. (B) Change in distinct PWM-model identities relative to Col-0; positive and negative values indicate gains and losses. (C) Percentage identity to the 49 bp Col-0 reference. Labels give the substitution number; non-reference variants carried one to four substitutions (91.8–98.0% identity). (D) Illustrative JASPAR logos for HSFC1 MA1667.2 (upper) and DOF1.8 MA0981.2 (lower); nucleotide heights show information content in bits. HSF-, DOF- and AP2/ERF-family PWM-model summaries are provided in Table S13; predictions do not establish binding or regulation *in vivo*.

To examine local sequence variation, we compared non-Col-0 candidate sequences with a single Col-0 reference seed (Fig. 6; Table S12). The displayed variants carried one to four substitutions and therefore retained 91.8–98.0% identity across the 49 bp seed (Fig. 7C). Differences occurred both within and outside the canonical HSE-like units. Because the search allowed no more than four mismatches to the Col-0 seed, these identity values describe only the detected candidate set and do not exclude more divergent *ONSEN*-related regions.

Most displayed variants retained above-threshold HSF-family predicted motif compatibility, whereas changes in other transcription-factor families were more frequent (Fig. 7A,B; Table S13). Fig. 7A and Table S13 report distinct PWM-model identities, not independent binding sites. The clearest recurrent shift was an increase in predicted DOF-family PWM models. Cvi-derived variants showed the largest reduction in distinct HSF models and increases of up to six DOF-family models, whereas the largest DOF gains (seven models) occurred in variants from An-1, Eri-1, Kyo and Sha. Fig. 7D provides representative HSF-family (upper) and DOF-family (lower) JASPAR sequence logos; nucleotide heights show information content in bits. These logos illustrate PWM sequence preferences and do not indicate transcription-factor occupancy *in vivo*.

## DISCUSSION

By integrating local sequence-design controls with genome-wide and accession-level analyses, we identify *ONSEN* terminal regions as a distinctive TE regulatory landscape: unusually dense and distributed non-redundant HSF-compatible sequence features coexist with elevated basal CHH methylation relative to non-*ONSEN* LTR retrotransposons and a pronounced heat-associated RNA-seq response, while detected natural variants generally preserve HSF compatibility and alter predicted DOF-family composition. These findings extend previous work on HSF-mediated *ONSEN* transcription and DNA-methylation-dependent silencing by placing local HSE sequence, terminal-region architecture, basal chromatin context and natural variation within one quantitatively controlled framework (Ito et al., 2011; Cavrak et al., 2014; Matsunaga et al., 2015; Hayashi et al., 2020; Nozawa et al., 2021). The analyses remain descriptive and do not establish that any feature, alone or in combination, is necessary or sufficient for *ONSEN* activation.

The designed-sequence analysis is most informative as an HSF-focused, case-specific control. Disruption of all five HSE cores eliminated predicted HSF-family motif compatibility in the selected design and every valid exact-GC alternative under the imposed constraints. Collateral changes predicted for other transcription-factor families depended on the exact substitutions and nucleotide composition and therefore support no general biological inference. More broadly, motif-disruption constructs should be evaluated against alternative sequence designs and composition-matched controls.

The genome-wide analyses showed that HSF-rich predicted sequence architecture is characteristic of *ONSEN* terminal candidate windows. Enrichment over the strict non-*ONSEN* TE background persisted across relative PWM-score thresholds of 0.80–0.95 and was reproduced by threshold-independent regional score summaries. Although a small number of non-*ONSEN* retrotransposons, particularly *ATHILA4A* elements, also exhibited elevated HSF motif density, *ONSEN* terminal candidate windows remained among the most strongly enriched TE regions. These results establish robust predicted HSF compatibility across *ONSEN* terminal regions but do not demonstrate HSF occupancy or causal regulatory activity *in vivo*.

The non-redundant HSF-coordinate analysis further clarifies the interpretation of motif density. Collapsing duplicate PWM-model/strand predictions at identical start–end coordinates reduced repeated counting while preserving 33–49 distinct coordinate placements in every *ONSEN* terminal window. The stricter overlap-merging sensitivity analysis reduced these values to 7–10 broader HSF-compatible regions per window. Agreement between these metrics shows that predicted HSF-rich architecture is distributed across the terminal candidate windows rather than arising from repeated counting of related PWM models at identical coordinates.

The methylation analysis adds an important biological contrast: *ONSEN* terminal regions carried selectively higher basal CHH methylation than a direct, annotation-defined non-*ONSEN* LTR-retrotransposon comparator while retaining strong HSF-compatible architecture. This coexistence places heat-responsive sequence potential within a repressive basal chromatin environment. Direct correlation between methylation change and transcription was not possible because the public methylome was generated from unstressed Col-0 leaves, whereas the RNA-seq data generated in this study used non-stressed and heat-treated whole-seedling samples. In a 37°C, 24-h experiment, Nozawa et al. (2021) reported no overall CHH hypermethylation at wild-type *ONSEN* after heat, despite changes in H3K9me2 and strong *ONSEN* transcription. A separate public bisulphite-sequencing dataset (GSE223850) profiles shoot-apical-meristem stem-cell nuclei 3 weeks after a 1-day 37°C exposure and identifies persistent heat-associated methylation changes, especially in the CHG context (Nguyen et al., 2025). Because that dataset differs in cell type, transgenic reporter background and recovery interval, treating it as paired with the analysed RNA-seq dataset would confound rather than clarify the comparison. We therefore interpret Fig. 4 as a basal-context analysis rather than a test of heat-induced methylation dynamics.

The RNA-seq analysis independently shows a broad heat response among genes and TEs, with all eight canonical *ONSEN* loci classified as upregulated in the global TE analysis. The multimapping-aware analysis further showed that heat-associated read accumulation was concentrated over *ONSEN* terminal candidate windows, while most HSF-rich non-*ONSEN* TE outliers showed little comparable increase. Because repetitive reads cannot be assigned unambiguously to individual copies, these results support an association between *ONSEN* terminal architecture and heat-responsive transcription but do not establish copy-specific expression or causal HSF occupancy.

Natural variation revealed a second robust pattern: within the detected candidate set, most *ONSEN*-like HSE-seed variants retained HSF-family motif compatibility while predicted DOF-family composition varied. This pattern indicates preservation of the core HSF-compatible sequence alongside diversification of overlapping motif composition. The recurrent DOF-family changes are therefore notable as candidate accession-dependent alterations in auxiliary cis-regulatory potential superimposed on a largely retained HSF-compatible core; they do not establish DOF-family binding or regulatory function. The Col-0-derived seed search necessarily enriches for Col-0-like sequences and may miss strongly diverged *ONSEN*-related regions; therefore, the result applies to the detected candidate set rather than establishing universal conservation across all natural *ONSEN* copies.

Published accession-specific studies provide additional biological context. Masuda et al. (2017) and Nozawa et al. (2022) documented variation in *ONSEN* copy number, methylation and heat-induced expression, including strong *ONSEN* induction and reduced CHH methylation in Kyoto. Xu et al. (2024) analysed control and heat RNA-seq in Col-0, Ler-1 and Cvi-0 and resolved substantial accession- and copy-dependent transcriptional variation. Because the published experiments differ in accession set, treatment, tissue, annotation and sequencing strategy, we used them for contextual comparison rather than combining them quantitatively with our seed-based motif survey.

Several limitations should be considered. First, motif analysis predicts sequence compatibility and does not demonstrate binding or regulatory activity *in vivo*. Second, repetitive *ONSEN* reads limit copy-specific RNA-seq interpretation. Third, the fixed 800 bp windows are operational terminal regions from a historically curated eight-locus set, not experimentally confirmed exact LTR boundaries or updated copy-integrity calls. Fourth, the accession survey used a Col-0-derived seed and can underestimate divergent *ONSEN*-related architectures. Fifth, methylation and RNA-seq were not measured in matched samples, precluding a direct test of heat-associated methylation-expression coupling. Finally, the predicted DOF-family shifts require experimental validation.

Overall, this study provides a quantitatively controlled map of *ONSEN* terminal-region regulatory architecture. The central advance is that *ONSEN* terminal candidate windows combine exceptionally strong, non-redundant HSF-compatible sequence features with elevated basal CHH methylation relative to other LTR retrotransposons and a marked heat-associated RNA-seq response, while detected natural variants generally preserve HSF compatibility and diversify overlapping motif composition. These associations do not establish a causal multilayer mechanism, but they substantially narrow the set of sequence and chromatin features to be tested through HSF-occupancy assays, reporter mutagenesis and accession-specific functional experiments.

## MATERIALS AND METHODS

### Plant materials and heat treatment

*A. thaliana* (L.) Heynh. accession Col-0 (ABRC stock CS60000) was used for all RNA-seq experiments. Seeds were surface sterilised and sown on half-strength Murashige and Skoog medium (Wako Pure Chemical Industries, Japan) supplemented with 0.8% (w/v) agar. After stratification at 4°C in darkness for 2 days, seedlings were grown for 7 days at 21°C under continuous white light (approximately 100 μmol m^−2^ s^−1^).

For heat treatment, plates containing 7-day-old seedlings were sealed with Parafilm and incubated at 37°C for 24 h. Control seedlings were maintained under identical conditions at 21°C. Whole seedlings were harvested immediately after treatment and frozen in liquid nitrogen. Three independent biological replicates were collected for each treatment.

### RNA extraction and RNA-seq

Total RNA was extracted from whole seedlings using the RNeasy Plant Mini Kit (Qiagen, Hilden, Germany) according to the manufacturer's instructions, including on-column DNase digestion. RNA quality and concentration were assessed using a NanoDrop 2000 spectrophotometer (Thermo Fisher Scientific).

Library preparation and sequencing were performed by HaploX Japan (Osaka, Japan). Poly(A)+ RNA was enriched, strand-specific libraries were prepared using standard Illumina protocols, and paired-end sequencing (2×150 bp) was carried out on an Illumina NovaSeq platform. Approximately 20 million paired-end reads were generated for each biological replicate. Adapter-trimmed FASTQ files supplied by the sequencing provider were used for all downstream analyses.

### *ONSEN* HSE sequence selection

A representative 49 bp sequence containing clustered *ONSEN* HSE-like units was selected from the LTR of a previously characterised *ONSEN* copy (Fig. 1).

Native sequence (49 bp): 5′-TCCTTAAAAGATTCTAGAAATTTTCTAGAATCATCTTCCACCTCCTTAA-3′.

*In silico*-designed HSE-disrupted sequence (49 bp): 5′-TCCTTAAAAGAGGCTACCGATTGGCTACCGTCATCGGCCACCTCCTTAA-3′.

An HSE-disrupted sequence of identical length was designed in silico by changing the TTC cores of the three nTTCn units to GGC and the GAA cores of the two nGAAn units to CCG. This introduced 12 substitutions at positions 12, 13, 17, 18, 19, 23, 24, 28, 29, 30, 36 and 37 (1-based coordinates), while preserving all non-targeted positions. GC content increased from 30.61% (15/49 nucleotides) to 51.02% (25/49 nucleotides). A custom scanner based on canonical nGAAn and nTTCn sequences confirmed that no five-base HSE-like units remained. The sequence was used only as an *in silico* perturbation; robustness was evaluated using the constrained-mutant analyses described in the following sections.

### Reference genome, genome annotation and motif databases

The *A. thaliana* TAIR10 reference genome and Ensembl Plants (release 63) annotation were used for genome-based analyses (Lamesch et al., 2012). From the historically established eight-locus Col-0 *ONSEN* set, fixed 800 bp windows were extracted from both terminal ends, yielding 16 terminal candidate windows used consistently for motif, methylation and RNA-seq analyses. These operational windows were not treated as experimentally validated exact LTR boundaries or updated copy-integrity calls. Genome-wide background analyses used TAIR10 TE annotations after excluding gene-associated features. Motif analyses used the JASPAR 2024 and 2026 CORE Plant collections (Rauluseviciute et al., 2024; Ovek Baydar et al., 2026). HSF-focused analyses used the ten *Arabidopsis* HSF-family models available in JASPAR 2026; because no HSFA2-specific model was available, results were interpreted at the HSF-family level.

### Motif analysis

Position frequency matrices were converted to PWMs using a pseudocount of 0.8 and equal nucleotide background frequencies (0.25) and then scanned on both strands with custom R scripts. Relative scores were calculated from each matrix's theoretical minimum and maximum. All above-threshold predictions were mapped to 1-based forward-sequence coordinates. The principal count metric collapsed duplicate PWM-model/strand predictions with identical start and end coordinates; predictions with different coordinates remained separate. Native-versus-designed model-level comparisons used the highest-scoring occurrence of each distinct PWM model.

A relative PWM score of 0.85 was the primary high-confidence cut-off. Genome-wide *ONSEN*-versus-background comparisons were also evaluated at 0.80, 0.90 and 0.95 and with continuous regional score summaries.

For principal non-redundant HSF motif-coordinate placements, predictions passing the selected score threshold were mapped to forward-sequence coordinates, and duplicates with identical 1-based start–end coordinates were collapsed across PWM models and strands. As a stricter sensitivity analysis, HSF intervals were merged transitively when they overlapped by at least one nucleotide; adjacent non-overlapping intervals remained separate. Neither metric represents experimentally verified HSF occupancy.

For comparisons of native versus HSE-disrupted sequences, motif models were classified as lost, gained, retained, strengthened, weakened or shifted according to changes in their best relative score and position. Models were grouped into transcription-factor families using JASPAR annotations and manual curation.

### Constrained-mutant sensitivity analysis

The robustness of HSF-family motif loss was tested with a separately validated family-focused set of 112 PWM models. Sequences were scanned on both strands at relative PWM-score thresholds of 0.80, 0.85, 0.90 and 0.95 (primary cut-off 0.85). Only the HSF-family robustness metric was carried forward for interpretation.

Using random seed 20260713, we generated 5000 unique constrained mutants. Exactly 12 of the 15 central positions in the five native HSE-like cores were substituted. Valid sequences disrupted all five cores, contained no canonical nTTCn or nGAAn unit anywhere in the 49 bp sequence, preserved every position outside the 15 core positions and were unique. GC content was unconstrained.

All valid constrained sequences matching the designed sequence's GC content (25/49 nucleotides; 51.02%) were also enumerated exhaustively. The constraints yielded 5120 exact-GC designs: the original design and 5119 alternatives. Non-redundant HSF motif-coordinate placements were summarised at the primary threshold. The variable-GC comparators contained only zero or one HSF prediction per sequence, and every exact-GC design contained zero; therefore, exact-coordinate deduplication could not change these distributions.

### Genome-wide analysis of *ONSEN* terminal candidate windows

The 16 fixed 800 bp *ONSEN* terminal candidate windows described above were used throughout the motif, methylation and RNA-seq candidate-region analyses. HSF motif density was calculated as the number of non-redundant exact-coordinate placements per kilobase. Strict overlap-merged HSF regions were retained as a sensitivity metric.

*ONSEN* windows were compared with a strict TAIR10 TE-only background comprising transposable_element, transposon_fragment and transposable_element_gene features after exclusion of gene-associated annotations.

After final annotation and coordinate harmonisation, the 16 *ONSEN* windows and 1930 strict non-*ONSEN* background regions were rescanned at relative-score cut-offs of 0.80, 0.85, 0.90 and 0.95 using the same ten HSF models and normalisation by sequence length. Continuous metrics were the maximum HSF score, mean of the five highest scores and median of the ten per-model maxima. Groups were compared using two-sided Wilcoxon rank-sum tests, with BH correction within related comparison sets and Cliff's delta as the effect size.

### DNA methylation analysis

Whole-genome bisulphite-sequencing data for unstressed Col-0 leaves were obtained from GEO GSE43857 (GSM1085222; Kawakatsu et al., 2016). For direct comparison with other LTR retrotransposons, annotation-defined non-*ONSEN* LTR retrotransposons were represented by fixed 800 bp terminal candidate windows at both element ends. The principal analysis aggregated methylated and total base counts across terminal windows for each element/copy, thereby avoiding pseudoreplication from paired termini. This yielded 779 non-*ONSEN* LTR-retrotransposon elements and six, six and eight covered *ONSEN* copies for CG, CHG and CHH, respectively. Window-level and broad ordinary-TE comparisons were retained as sensitivity analyses.

Weighted methylation was calculated as 100× the sum of methylated-base counts divided by the sum of total-base counts within each analysis unit and context. Regions lacking covered cytosines were omitted. Regions lacking covered cytosines were omitted. Copy/element-level *ONSEN* and non-*ONSEN* LTR retrotransposons were compared within each context using two-sided Wilcoxon rank-sum tests with BH correction across contexts. The window-level analysis used ten, ten and 16 *ONSEN* terminal windows and 1549, 1549 and 1552 non-*ONSEN* LTR-retrotransposon terminal windows for CG, CHG and CHH, respectively. Locus-level displays used the fixed eight-region HSF-rich non-*ONSEN* TE subset listed in Table S8D.

### RNA-seq analysis

Adapter-trimmed paired-end RNA-seq reads were aligned to the complete TAIR10 reference, including chromosomes 1–5 and the mitochondrial and plastid sequences, using Rsubread. Gene counts were generated from exon annotation and summarised by gene with featureCounts (Liao et al., 2014); all 32,833 annotated genes were retained, and non-stressed versus heat-stressed samples were analysed with DESeq2 (Love et al., 2014). DESeq2 returned model estimates for 25,912 genes, raw *P*-values for 25,911 and non-missing BH-adjusted *P*-values for 23,922 after independent filtering. For the global TE analysis, all 31,189 Araport11 *transposable_element* loci were retained, multimapping and overlapping reads were assigned fractionally, and 2101 loci passing expression filtering were analysed with limma-voom. Upregulated and downregulated calls required BH-adjusted *P*<0.05 and an absolute log2 fold change of at least 1. Features without model estimates, as well as genes whose BH-adjusted *P*-value was unavailable after independent filtering, were retained with missing entries in Table S9. Gene and TE volcano plots are shown in Fig. 5D,E.

For Fig. 5A, BH-adjusted DESeq2 *P*-values were used for the selected heat-responsive genes. For visualisation only, relative expression was calculated as 2^(VST_sample−mean VST_NS)^ from variance-stabilising transformation (VST) values, setting the mean non-stressed baseline to 1. *ONSEN* terminal windows and the fixed HSF-rich non-*ONSEN* TE subset were quantified separately using a custom Simplified Annotation Format (SAF) annotation. Paired-end counting used fractional assignment of multimapping reads and multiple-overlap counting. Signals were normalised to assigned gene reads and expressed as fractional CPM mapped reads.

For Fig. 5B, fractional CPM was summed across the 16 *ONSEN* terminal candidate windows for each biological replicate and compared between conditions using two-sided Welch's *t*-tests; per-window summaries and the corresponding Wilcoxon result are reported in Table S10. For Fig. 5C, the horizontal coordinate was log2(mean HS CPM+1), whereas the point area encoded max{0, log2[(mean HS CPM+0.05)/(mean NS CPM+0.05)]}. Thus, the +0.05 pseudocount was used only for the descriptive fold-change measure, and negative values were truncated to zero only for point sizes. The panel contains the 16 curated *ONSEN* terminal windows, two additional *ATCOPIA78* candidate regions and the fixed eight-region HSF-rich non-*ONSEN* TE subset. Because of multimapping, these measurements represent the candidate-window signal rather than copy-specific expression.

### Natural accession analysis

Chromosome-level assemblies from An-1, C24, Cvi-0, Eri-1, Kyo, Ler-1 and Sha were analysed with Col-0 (Jiao and Schneeberger, 2020). The 49 bp Col-0 HSE-containing sequence was used to recover candidates with up to four mismatches, together with surrounding LTR sequence. Candidates were evaluated with the same HSE and motif pipeline. Variants were compared with one Col-0 reference seed to separate accession polymorphism from Col-0 copy variation. Substitution number was the mismatch count, and identity was calculated as 100×(49−mismatches)/49. Neighbouring HSE-containing regions were classified as paired HSE/LTR candidates when their spacing matched the LTR distances of curated full-length Col-0 *ONSEN* elements. These were treated as structural proxies, not definitive copy-number estimates.

Motif changes were summarised by transcription factor family. These family-level PWM-model summaries are analytically distinct from the ten-model *Arabidopsis* HSF exact-coordinate analyses described above. Fig. 7A and Table S13 report distinct JASPAR PWM-model identities with at least one above-threshold match, rather than independent motif-coordinate placements or binding sites. Fig. 7D shows illustrative information-content logos for HSFC1 MA1667.2 (upper) and DOF1.8 MA0981.2 (lower); these were not used for the JASPAR 2026 HSFC1 MA1667.3 counts in the primary HSF scan. Predictions do not demonstrate *in vivo* binding.

### Statistical analysis

Analyses were performed in R 4.4.3 using custom scripts, with dplyr, tidyr and readr for data handling and ggplot2 for figures. Sample sizes for sequence and genomic analyses denote computational units. Unless stated otherwise, two-group comparisons used two-sided Wilcoxon rank-sum or Welch's *t*-tests, with BH correction for multiple comparisons. Genome-wide motif effect sizes used Cliff's delta. Constrained-mutant analyses used plus-one empirical upper-tail probabilities and Spearman correlation. Analysis-specific tests and sample sizes are given in the relevant subsection, legend or supplementary table.

RNA-seq summaries are presented as means±s.e.m.; genomic distributions use the summaries specified in each figure legend. Statistical significance was *P*<0.05 unless a stricter adjusted threshold is stated. No formal power calculation was performed. Welch's *t*-tests were used without assuming equal variances, and Wilcoxon rank-sum tests were used as non-parametric comparisons. No samples were excluded from the analyses, and experiments were not randomised or blinded.

### Declaration of generative-AI- and AI-assisted technologies

OpenAI ChatGPT/Codex (GPT-5) was used during manuscript revision for code troubleshooting and data-analysis-script optimisation. It was not used to generate experimental data or figure images. All AI-assisted output was reviewed and verified by the authors, who take full responsibility for the content of the manuscript.

## Supplementary Material



10.1242/biolopen.062799_sup1 Supplementary information

Table S1. Native versus in-silico-designed HSE-disrupted 49-bp *ONSEN* sequence: PWM-model effects and family-specific non-redundant motif-coordinate placements.

Table S2. *Arabidopsis* HSF-family PWM models falling below the relative PWM-score threshold of 0.85 in the in-silico-designed HSE-disrupted 49-bp *ONSEN* sequence.

Table S3. HSF-focused constrained-mutant and exact-GC robustness control for the in-silico-designed 49-bp *ONSEN* HSE-disrupted sequence.

Table S4. Non-redundant *Arabidopsis* HSF-family motif-coordinate summary and density across Col-0 *ONSEN* terminal candidate windows.

Table S5. Principal non-redundant HSF motif-coordinate placements and strict overlap-merged HSF regions in the native and in-silico-designed HSE-disrupted 49-bp sequences and Col-0 *ONSEN* terminal candidate windows.

Table S6. Strict non-*ONSEN* transposable-element background comparison of non-redundant HSF motif-coordinate placement density at relative PWM-score thresholds of 0.85 and 0.90.

Table S7. Threshold sensitivity and continuous-score analysis of non-redundant HSF-family motif enrichment in Col-0 *ONSEN* terminal candidate windows relative to a strict non-*ONSEN* transposable-element background.

Table S8. Direct methylation comparison between *ONSEN* terminal candidate regions and annotation-defined non-*ONSEN* LTR-retrotransposon terminal regions, with window-level and broad-TE sensitivity analyses.

Table S9. Genome-wide gene and transposable-element differential expression in Col-0 after 24-h 37°C heat stress.

Table S10. Heat-associated RNA-seq signal across curated *ONSEN* terminal candidate windows, two additional *ATCOPIA78* candidate regions and the fixed HSF-rich non-*ONSEN* TE subset.

Table S11. Natural-accession *ONSEN*-like candidate-window abundance, non-redundant HSF motif-coordinate density and paired HSE/LTR structural-proxy summary.

Table S12. Natural 49-bp *ONSEN*-like HSE seed-variant accession summary.

Table S13. Natural seed-variant transcription-factor-family PWM-model summary.
