# Supplementary material for: Heat-responsive ONSEN long terminal repeats integrate heat shock factor motifs, DNA methylation and natural sequence variation in Arabidopsis
Source: Biol Open. 2026 Sep 11;15(9):bio062799. doi: 10.1242/bio.062799 (PMC13602373; doi:10.1242/bio.062799)
Supplement: Supplementary file 1 [file biolopen-15-062799-s1.pdf]

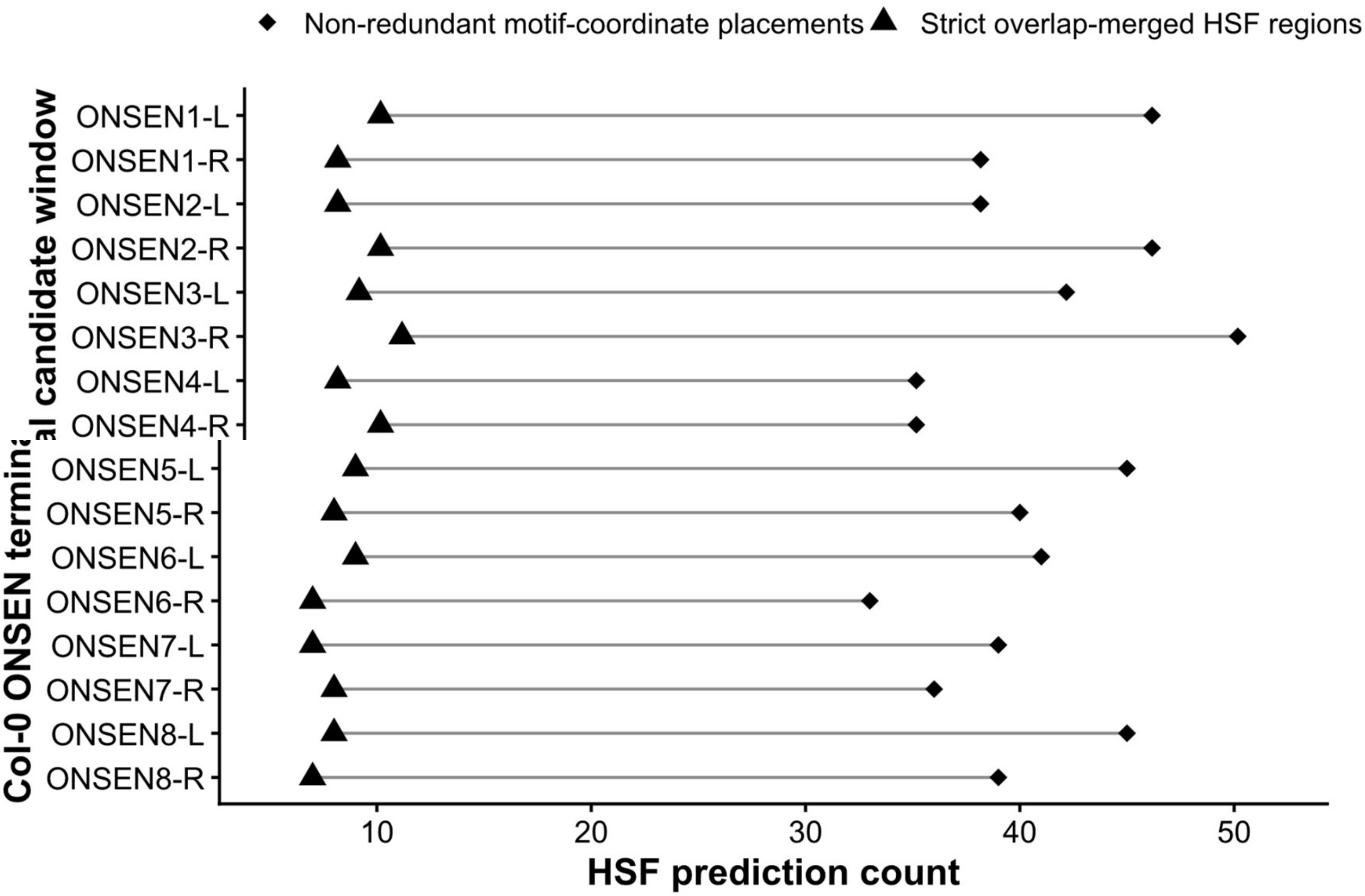

Fig. S1. Principal non-redundant HSF motif-coordinate placements and strict overlap-merged HSF regions in Col-0 *ONSEN* terminal candidate windows. HSF predictions were filtered at relative PWM score ≥0.85. Diamonds indicate exact-coordinate placements after duplicate PWM-model/strand predictions with identical 1-based start–end coordinates were collapsed. Triangles indicate the stricter sensitivity metric obtained by transitively merging intervals that overlapped by at least one nucleotide. Connected symbols represent the same 800-bp terminal candidate window. Both metrics indicate predicted sequence compatibility and not independent HSF-binding events or occupancy *in vivo*.

Biology Open • Supplementary information

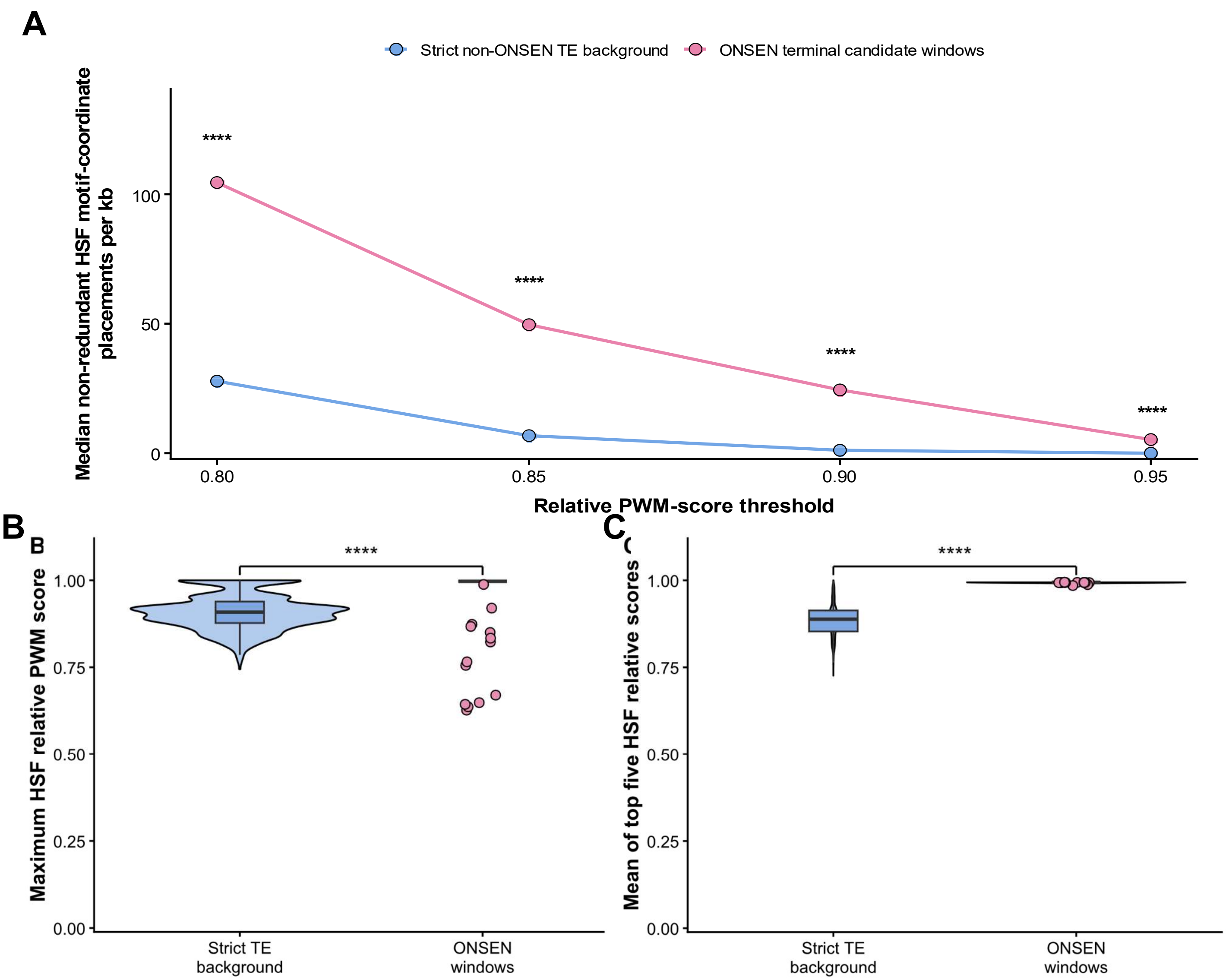

Fig. S2. HSF-family motif enrichment in *ONSEN* terminal candidate windows is robust to score threshold and continuous-score analysis. (A) Median density of non-redundant HSF motif-coordinate placements in sixteen Col-0 *ONSEN* 800-bp terminal candidate windows and 1,930 strict non-*ONSEN* TE regions at relative PWM-score thresholds of 0.80, 0.85, 0.90 and 0.95. Asterisks indicate the *ONSEN*-versus-background comparisons at each threshold. (B) Distribution of the maximum HSF relative PWM score per region. (C) Distribution of the mean of the five highest HSF relative PWM scores per region. Violin and box plots show the strict TE background, and individual *ONSEN* regions are shown as pink points. Brackets indicate group comparisons; ****,BH-adjusted two-sided Wilcoxon P<0.0001. Exact statistics, including the median of ten per-model maximum scores and Cliff's delta effect sizes, are provided in Table S7.

Biology Open • Supplementary information

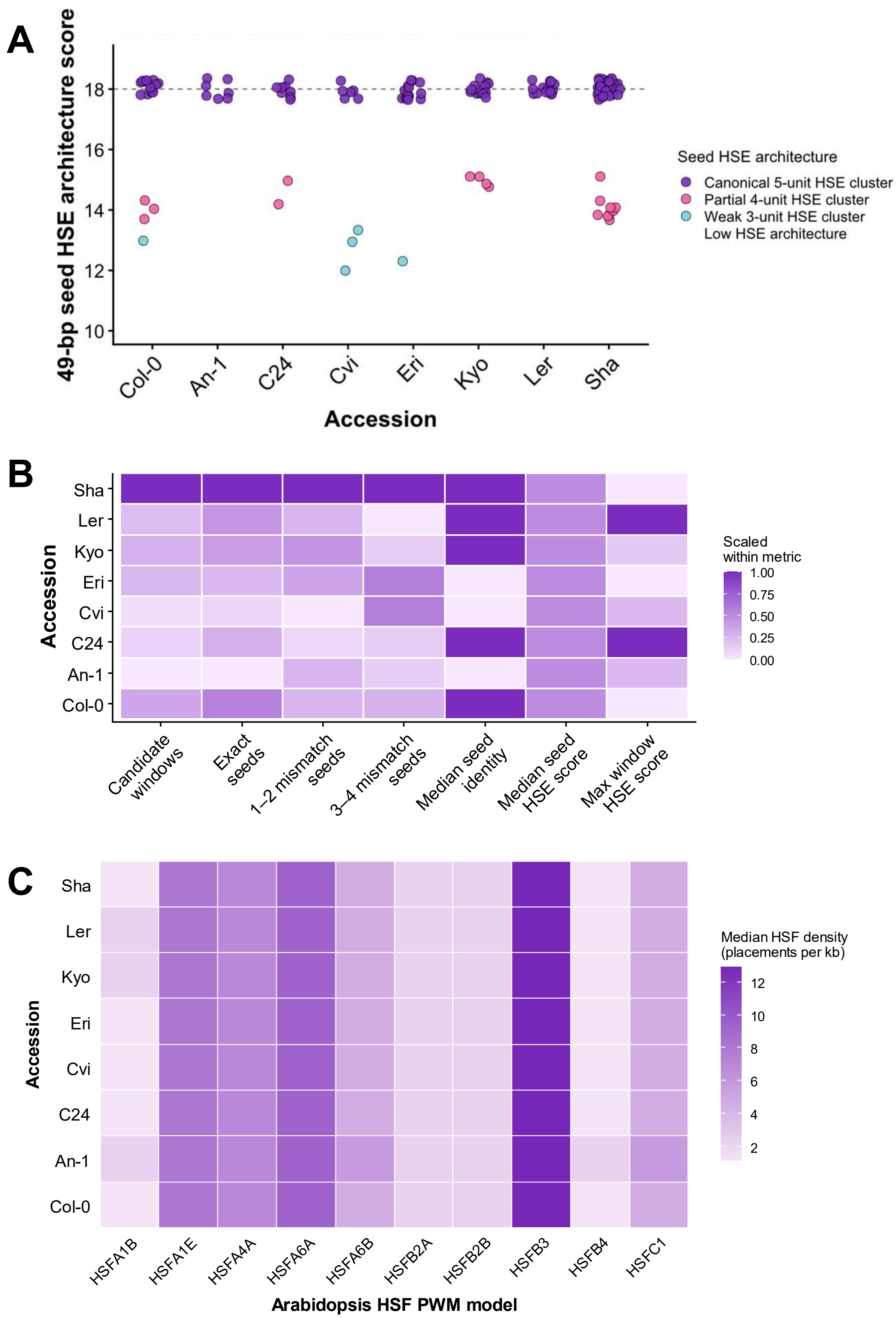

Biology Open • Supplementary information

Fig. S3. Additional summaries of natural sequence variation and HSF-family motif compatibility across *Arabidopsis* accessions.(A) HSE architecture scores of 49-bp *ONSEN*-like HSE candidate sequences identified in natural *Arabidopsis thaliana* accessions. Candidate sequences were classified as canonical five-unit, partial four-unit, weak three-unit or low HSE architectures according to the number of canonical HSE-like units detected. The dashed line indicates the canonical five-unit HSE architecture of the Col-0 reference sequence. (B) Scaled summary of accession-level *ONSEN*-like HSE candidate metrics. Variables include the number of candidate windows, exact sequence matches, sequences with one to two or three to four mismatches, median sequence identity, median HSE architecture score and maximum HSE architecture score. Each variable was independently scaled to facilitate comparison among accessions. (C) Median non-redundant HSF motif-coordinate placement density across *ONSEN*-like candidate regions in each accession. Densities were calculated using the retained *Arabidopsis* HSF-family PWM models from the JASPAR 2026 CORE Plant database. Predicted motif densities represent sequence compatibility and do not indicate direct transcription-factor binding *in vivo*.

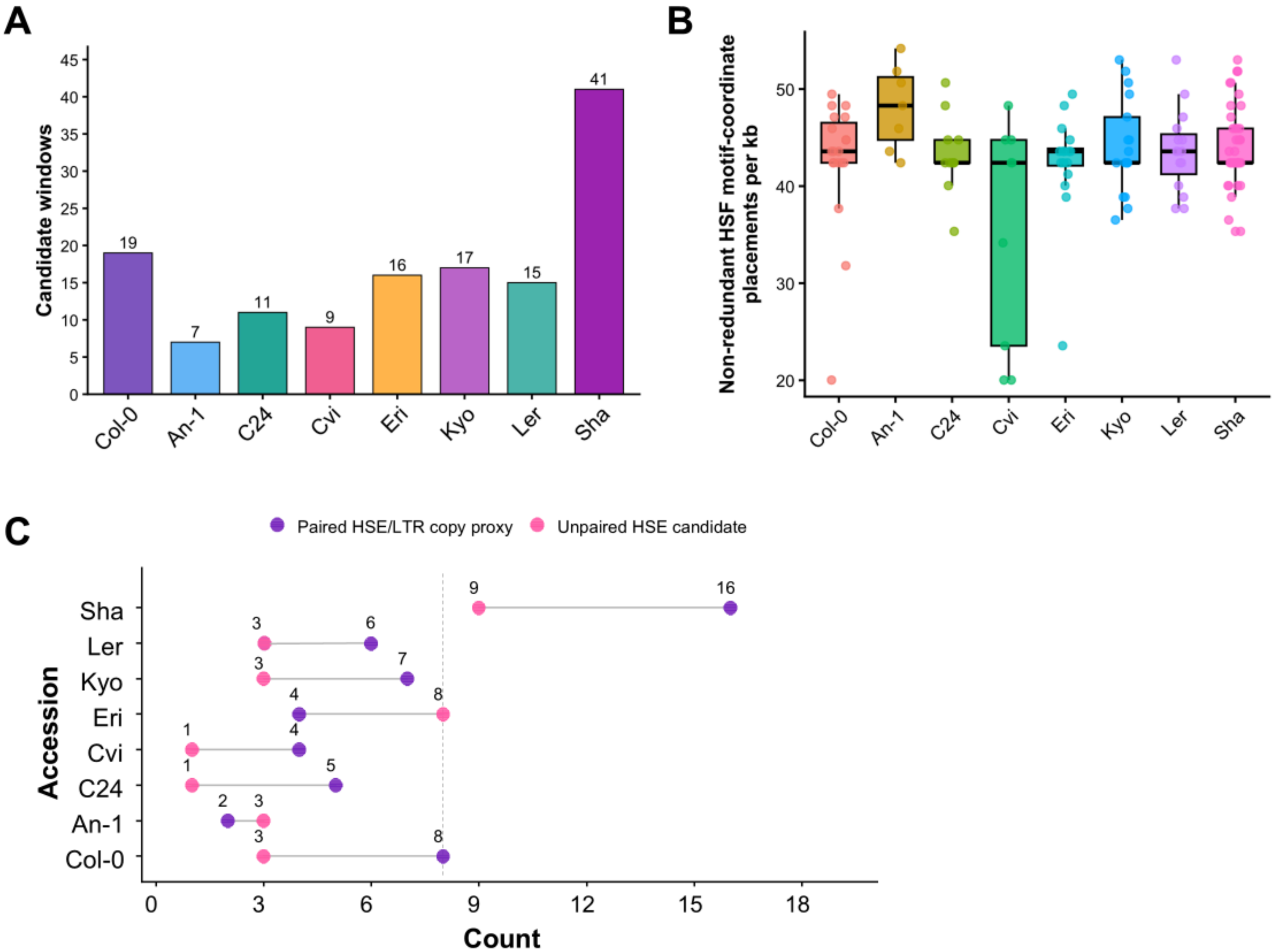

Fig. S4. Natural-accession variation in *ONSEN*-like HSE candidate abundance and HSF-family motif composition. (A) Number of *ONSEN*-like HSE candidate windows identified on the main chromosomes of eight *Arabidopsis thaliana* accessions using the 49-bp Col-0 *ONSEN* HSE sequence as the query, allowing up to four nucleotide mismatches. (B) Distribution of non-redundant HSF motif-coordinate placement density among accession-specific *ONSEN*-like candidate windows. Boxplots show the median, interquartile range (IQR) and 1.5×IQR whiskers; points represent individual candidate windows. (C) Accession-level summary of paired HSE/LTR structural proxies and unpaired HSE candidate windows. Candidate windows were classified as paired when the distance between neighbouring HSE-containing regions fell within the LTR-to-LTR spacing range observed for curated full-length *ONSEN* (*ATCOPIA78*) elements in Col-0. Unpaired counts equal total candidate windows minus two windows per paired proxy; for Ler, 15 windows comprise six paired proxies and three unpaired windows. These values are structural proxies and should not be interpreted as definitive *ONSEN* copy-number estimates. Counts are provided in Table S11.

Table S1. Native versus in-silico-designed HSE-disrupted 49-bp *ONSEN* sequence: PWM-model effects and family-specific non-redundant motif-coordinate placements.

Available for download at
https://journals.biologists.com/bio/article-lookup/doi/10.1242/bio.062799#supplementary-data

Table S2. *Arabidopsis* HSF-family PWM models falling below the relative PWM-score threshold of 0.85 in the in-silico-designed HSE-disrupted 49-bp *ONSEN* sequence.

Available for download at
https://journals.biologists.com/bio/article-lookup/doi/10.1242/bio.062799#supplementary-data

Table S3. HSF-focused constrained-mutant and exact-GC robustness control for the in-silico-designed 49-bp *ONSEN* HSE-disrupted sequence.

Available for download at
https://journals.biologists.com/bio/article-lookup/doi/10.1242/bio.062799#supplementary-data

Table S4. Non-redundant *Arabidopsis* HSF-family motif-coordinate summary and density across Col-0 *ONSEN* terminal candidate windows.

Available for download at
https://journals.biologists.com/bio/article-lookup/doi/10.1242/bio.062799#supplementary-data

Table S5. Principal non-redundant HSF motif-coordinate placements and strict overlap-merged HSF regions in the native and in-silico-designed HSE-disrupted 49-bp sequences and Col-0 *ONSEN* terminal candidate windows.

Available for download at
https://journals.biologists.com/bio/article-lookup/doi/10.1242/bio.062799#supplementary-data

Table S6. Strict non-*ONSEN* transposable-element background comparison of non-redundant HSF motif-coordinate placement density at relative PWM-score thresholds of 0.85 and 0.90.

Available for download at
https://journals.biologists.com/bio/article-lookup/doi/10.1242/bio.062799#supplementary-data

Table S7. Threshold sensitivity and continuous-score analysis of non-redundant HSF-family motif enrichment in Col-0 *ONSEN* terminal candidate windows relative to a strict non-*ONSEN* transposable-element background.

Available for download at
https://journals.biologists.com/bio/article-lookup/doi/10.1242/bio.062799#supplementary-data

Table S8. Direct methylation comparison between *ONSEN* terminal candidate regions and annotation-defined non-*ONSEN* LTR-retrotransposon terminal regions, with window-level and broad-TE sensitivity analyses.

Available for download at
https://journals.biologists.com/bio/article-lookup/doi/10.1242/bio.062799#supplementary-data

Table S9. Genome-wide gene and transposable-element differential expression in Col-0 after 24-h 37°C heat stress.

Available for download at
https://journals.biologists.com/bio/article-lookup/doi/10.1242/bio.062799#supplementary-data

Table S10. Heat-associated RNA-seq signal across curated *ONSEN* terminal candidate windows, two additional *ATCOPIA78* candidate regions and the fixed HSF-rich non-*ONSEN* TE subset.

Available for download at
https://journals.biologists.com/bio/article-lookup/doi/10.1242/bio.062799#supplementary-data

Table S11. Natural-accession *ONSEN*-like candidate-window abundance, non-redundant HSF motif-coordinate density and paired HSE/LTR structural-proxy summary.

Available for download at
https://journals.biologists.com/bio/article-lookup/doi/10.1242/bio.062799#supplementary-data

Table S12. Natural 49-bp *ONSEN*-like HSE seed-variant accession summary.

Available for download at
https://journals.biologists.com/bio/article-lookup/doi/10.1242/bio.062799#supplementary-data

Table S13. Natural seed-variant transcription-factor-family PWM-model summary.

Available for download at
https://journals.biologists.com/bio/article-lookup/doi/10.1242/bio.062799#supplementary-data
