# Supplementary material for: Heat-responsive ONSEN long terminal repeats integrate heat shock factor motifs, DNA methylation and natural sequence variation in Arabidopsis
Source: Biol Open. 2026 Sep 11;15(9):bio062799. doi: 10.1242/bio.062799 (PMC13602373; doi:10.1242/bio.062799)
Supplement: Supplementary file 2 [file biolopen-15-062799_review_history.pdf]

Heat-responsive ONSEN long terminal repeats integrate heat shock factor motifs, DNA methylation and natural sequence variation in Arabidopsis

Baibhav R. Barbaruah, Rahmadani P. Airalangga and Hidetaka Ito
10.1242/bio.062799

Editor: Marie Monniaux

Review timeline

Original submission:	6 July 2026
Editorial decision:	10 July 2026
First revision received:	17 July 2026
Editorial decision:	5 August 2026
Second revision received:	17 August 2026
Accepted:	17 August 2026

Original submission

First decision letter

MS ID#: bio.062799

MS Title: Heat-responsive ONSEN long terminal repeats integrate heat shock factor motifs, DNA methylation and natural sequence variation in Arabidopsis

Authors: Baibhav R. Barbaruah, Rahmadani P. Airalangga and Hidetaka Ito

I have now reached a decision on the above manuscript.

The reviewer reports are shown at the bottom of this email.

As you will see, the reviewers raised a number of substantial criticisms that prevent me from accepting the paper at this stage.

They suggest, however, that a revised version might prove acceptable, if you can address their concerns. If you think that you can deal satisfactorily with the criticisms on revision, I would be pleased to see a revised manuscript. We would then return it to the reviewers.

Please provide a point-by-point answer to the reviewers comments, in particular regarding the details of how the mutant sequence has been generated, how motif scanning has been performed, and please provide a control for the DNA methylation analysis. Scripts and sequencing datasets should also be deposited.

At this stage, we also ask you to ensure your manuscript complies with our formatting guidelines. Provided you are able to fully address the referees' comments, we are positive about publication of your paper (we accept over 95% of revision submissions) and therefore hope you won't mind any extra work involved in reformatting your manuscript at this point.

© 2026. Published by The Company of Biologists under the terms of the Creative Commons Attribution License (https://creativecommons.org/licenses/by/4.0/).

Please upload both a 'clean' version of your Word file, along with a highlighted version clearly showing where you have made changes in the revised manuscript. Please avoid using 'Track changes' in Word files as these are lost in PDF conversion.

I should be grateful if you would also provide a point-by-point response detailing how you have dealt with the points raised by the reviewers in the 'Response to Reviewers' box. Please attend to all of the reviewers' comments. If you do not agree with any of their criticisms or suggestions please explain clearly why this is so.

Reviewer 1

Comments for the author

The manuscript by Barbaruah B.R. et al. investigates the regulatory architecture of Arabidopsis thaliana ONSEN transposable elements, which harbor heat shock elements. The authors combine transcription factor motif prediction to characterize HSF-binding motifs within ONSEN transposons with DNA methylation analyses to assess the epigenetic layer of regulation and RNA-seq experiments under control and heat-shock conditions.

The study addresses an interesting question regarding the evolution of cis-regulatory elements within transposable elements and provides a useful framework for considering the multilayered regulation of transposable element expression. I also appreciate the authors' emphasis on naturally occurring polymorphic TE insertions as a potential source of regulatory and phenotypic variation.

However, several aspects of the study require clarification or additional analyses before the conclusions can be fully supported. In particular, the design and interpretation of the engineered mutant sequence require substantially more detail, as one of the major conclusions of the manuscript depends on a single in silico-designed mutant. In addition, the rationale for the motif-scanning strategy, the choice of PWM score thresholds, and the interpretation of motif enrichment would benefit from further justification. Finally, several methodological details, including the availability of the custom analysis scripts and the deposition of the sequencing datasets, should be clarified to improve the reproducibility of the study.

My major comments are listed below.

L85-110: It is unclear whether the mutant sequence represents an experimentally generated mutant or an in silico-designed sequence. Please clarify this explicitly in both the Results and the Methods. In addition, please describe the rationale used to design the mutant sequence. Why were 12 substitutions introduced, and why was this number chosen? Later in the manuscript, authors show much smaller number of substitutions that occur in natural genomic sequences across different accessions. Which sequence properties were preserved during the design process? And how were the specific nucleotide substitutions selected?

The downstream analysis of the mutant sequence, and the conclusion that HSE disruption rewires the local cis-regulatory landscape through the gain of new transcription factor binding motifs, are heavily influenced by the design of the mutant itself. Because the sequence was intentionally engineered rather than generated through an unbiased mutational process (if I understood correctly), the observed gain and loss of TF-binding motifs cannot be considered an independent observation and depends on the particular set of nucleotide substitutions that was introduced. Therefore, it is difficult to conclude that disruption of an HSE results in an AP2/ERF-enriched regulatory landscape.
To support such conclusion, the authors should demonstrate that the observed pattern is robust across multiple independently generated HSE-disrupting mutant sequences designed under the same constraints. For example, the authors could generate an unbiased set of mutant sequences by systematically and randomly disrupting the canonical HSF archetypal binding motif. Alternatively, they could analyze natural sequence variation within ONSEN HSE regions across different Arabidopsis accessions used in this study, or for example using the 1001 Genomes dataset, to determine whether natural disruption of HSE motifs (within and between genomes) is consistently associated with the emergence of AP2/ERF or other specific transcription factor binding motifs.

© 2026. Published by The Company of Biologists under the terms of the Creative Commons Attribution License (https://creativecommons.org/licenses/by/4.0/).

Line 116: The HSF-family PWMs included in Jaspar are highly similar. Therefore, multiple motif models are likely to match the same genomic location. Reporting the total number of motifs may substantially overestimate the number of independent binding sites. Please clarify whether overlapping matches from different HSF models were counted separately. If so, I recommend also reporting the number of non-redundant genomic locations occupied by HSF-family motifs, which would provide a more biologically meaningful estimate of motif density.

Line 125: How were the PWM score threshold of 0.85 and 0.9 selected? Please provide the rationale for these cutoffs, as motif enrichment can be sensitive to the choice of threshold. In addition to reporting thresholded motif counts, it would be informative to show the genome-wide distribution of HSF-family PWM scores across ONSEN LTRs, non-ONSEN LTRs, other TE families, and non-ONSEN non-TE genomic regions. Such an analysis would help determine whether ONSEN LTRs exhibit a general shift toward higher HSF motif scores, rather than an enrichment that depends on specific score cutoffs. Continuous motif-scoring approaches (for example, using the monaLisa R package to annotate genomic regions with motif archetypes) could further strengthen the analysis by reducing its dependence on the selected thresholds.

Line 213: This section would fit better in the Discussion, as it does not present new data or results but rather compares the findings with the existing literature.

Figure 7: Consider including motif logos together with motif similarity scores (or another quantitative measure of motif similarity). This would help readers assess the degree of sequence conservation, the number of nucleotide substitutions, and the information content of the compared motifs.

Line 239: This conclusion appears overstated. The analysis is based on a single in silico-designed mutant generated by introducing 12 nucleotide substitutions specifically to abolish HSE motifs. Therefore, the observed gain of AP2/ERF-associated motifs is conditional on the particular mutational design and cannot be generalized as an inherent consequence of HSE disruption.

Methods: The manuscript refers to several custom R scripts for motif scanning and analysis. Please indicate whether these scripts will be made publicly available to ensure the reproducibility of the analyses.

Data availability: The manuscript does not appear to provide accession numbers for the generated RNA-seq datasets.

<u>Reviewer 2</u>

Comments for the author

In Arabidopsis thaliana, exposure to prolonged heat stress induces activation of stress responsive retrotransposons, including the ONSEN TY1/Copia retrotransposon. ONSEN features heat shock element (HSE)-like sequences within its long terminal repeats (LTRs). ONSEN is generally repressed by DNA methylation, however it has been shown that upon heat stress, the heat shock factor HSFA2 binds to HSE-like sequences and activates ONSEN expression.

In this manuscript, the authors address the regulatory potential of heat shock element (HSE)-like sequences across the ONSEN family. They start by comparing a representative 49-bp LTR sequence containing clustered HSE-like motifs in ONSEN with a mutant carrying 12 nucleotide substitutions within the HSE-rich region. Genome-wide motif scanning suggests that in the mutant 31 HS motifs are lost while new AP2/ERF-family motifs are acquired. Analysis of 16 LTRs from ONSEN elements showed that, compared with annotated TEs, ONSEN elements are generally more enriched for HS motifs. Analysis of publicly available methylomes from unstressed leaves shows that ONSEN sequences have generally high levels of DNA methylation, indicating that they are normally transcriptionally repressed. Analysis of RNA-seq datasets from seedlings exposed to 37°C for 24 h showed increased expression of HS genes and ONSEN TEs compared to non-stressed controls, curiously, HSF motif-containing TEs other than ONSEN were not upregulated.

© 2026. Published by The Company of Biologists under the terms of the Creative Commons Attribution License (https://creativecommons.org/licenses/by/4.0/).

Analysis of genome assemblies from 7 Arabidopsis populations showed that compared to the reference genome (Col-0), ONSEN elements retain high-confidence HSF-family motifs even though several nucleotide substitutions were observed. These substitutions seemed to affect the motifs of other TFs.This is a very interesting result, which, together with the fact that ONSEN LTRs are generally enriched in HSF-rich motifs, suggests that regulation by the HSF is constrained and potentially functionally important for ONSEN across Arabidopsis populations.

In summary, although the fact that ONSEN is regulated by heatshock had been previously shown, the authors provide an interesting analysis of the HSF motifs in ONSEN LTR, which could be used in experimental studies aimed at determining the role of these sequences in HS-dependent regulation and provide nice evidence that these motifs are constrained and could therefore be functionally important. However, no experimental evidence is provided to support their hypothesis that integration of conserved cis-regulatory architecture, epigenetic context and natural sequence variation, rather than an individual heat shock element alone, is necessary for ONSEN regulation. Regarding the epigenetic regulation, the authors only examine DNA methylation levels in non-stressed plants. In addition, they do not provide an analysis of RNA-seq data to show how different Arabidopsis populations respond to heat stress. Therefore, in my opinion, the analysis is not sufficiently developed to warrant publication in this journal and I would suggest submitting to a more specialized bioinformatic journal.

Comments:

A detailed description of the criteria used to generate the 12nt mutant within the HSE-rich region is missing in the materials and methods.

In Figure 3, the authors show that 16 LTR from 8 ONSEN elements present a high density of HSF motifs. How many ONSEN elements exist in Arabidopsis Col_0? And why choose only these elements for their analysis?

In Figure 4, they show the DNA methylation profiles of ONSEN LTR in non-stressed conditions and in Figure 5, the changes in ONSEN expression upon heat stress. Do the changes in transcription correlate with changes in DNA methylation? Analysis of DNA methylation upon heat stress is missing.

In Figure 6, the authors show that ONSEN HSE motifs are quite conserved across natural Arabidopsis populations. Do they have RNA-seq data on the different species to show how ONSEN TE respond to heat shock in the different populations?

Reviewer's Responses to Questions

Experimental quality
Are experiments performed using appropriate methods that will answer the question (or test the hypothesis or support the observations) posed by the authors? Is the right tool used for the job?
If 'No', please indicate reasons in Comments for Author box below.
Reviewer #1:

- Yes

Reviewer #2:

- Yes

Were the data analyzed using appropriate statistical tests?
If 'No', please indicate reasons in Comments for Author box below.
Reviewer #1:

- Yes

Reviewer #2:

- Yes

© 2026. Published by The Company of Biologists under the terms of the Creative Commons Attribution License (https://creativecommons.org/licenses/by/4.0/).

Reproducibility
Were experiments performed using adequate number of biological replicates?
If 'No', please indicate reasons in Comments for Author box below.
Reviewer #1:
- Yes

Reviewer #2:
- Yes

Is there sufficient raw data to assess the rigor of the analysis?
If 'No', please indicate reasons in Comments for Author box below.
Reviewer #1:
- Yes

Reviewer #2:
- Yes

Does the methods section provide sufficient detail to permit reproducibility?
If 'No', please indicate reasons in Comments for Author box below.
Reviewer #1:
- No

Reviewer #2:
- Yes

Completeness
Are the manuscript's conclusions supported by the data?
If 'No', please indicate reasons in Comments for Author box below.
Reviewer #1:
- No

Reviewer #2:
- No

Scholarship
Do the authors cite and discuss the merits of data that would argue for and against their conclusion?
If 'No', please indicate reasons in Comments for Author box below.
Reviewer #1:
- Yes

Reviewer #2:
- No

Does the manuscript title & abstract accurately reflect the contents of the manuscript, without hyperbole?
If 'No', please indicate reasons in Comments for Author box below.
Reviewer #1:
- Yes

Reviewer #2:
- No

© 2026. Published by The Company of Biologists under the terms of the Creative Commons Attribution License (https://creativecommons.org/licenses/by/4.0/).

First revision

<u>Author response to reviewers' comments</u>

Dear Dr Monniaux,

We thank you and the reviewers for your careful assessment of our manuscript. We have revised the manuscript extensively in response to the comments. The main changes include additional controls for sequence design, motif-score thresholds, PWM redundancy and methylation comparisons, together with a more cautious treatment of natural variation. We also expanded the Materials and Methods and data-availability sections, reorganised parts of the Results and Discussion, and revised statements that went beyond what the data support. We retained the title. In this context, 'integrate' refers to the combined analysis of sequence, methylation, transcriptomic and accession-level data, and not to a claim that these features are necessary or sufficient for *ONSEN* activation. Our responses are given below.

Overview of the major revisions

- Clarified that the 49-bp HSE-disrupted sequence was designed in silico, and reported the 12 substitutions, the five targeted HSE-like cores, the properties retained in the design and the change in GC content.
- Tested the sequence design using 5,000 constrained random mutants and all 5,120 valid exact-GC designs, allowing robust HSF loss to be distinguished from composition-dependent changes in other motif families.
- Added a non-redundant HSF-compatible-location analysis to distinguish overlapping PWM model-position hits from separate compatible sequence intervals.
- Repeated the *ONSEN*-versus-background comparison at relative PWM-score thresholds of 0.80, 0.85, 0.90 and 0.95, and added continuous regional-score summaries.
- Added 1,942 ordinary transposable elements as a methylation control and limited the interpretation to the basal, unstressed context represented by the public methylome.
- Clarified that the sixteen fixed 800-bp terminal candidate windows were taken from both ends of eight historically curated Col-0 *ONSEN* loci and are operational analysis regions, not experimentally confirmed exact LTR boundaries.
- Added percentage identity and substitution counts for the detected natural variants, together with representative HSF- and DOF-family motif logos.
- Moved the literature-based synthesis from the Results to the Discussion and revised the Abstract, Summary statement, Results, Discussion and figure legends to separate association from causation.
- Deposited the ordered workflow, configuration, provenance, validation procedures and supplementary outputs on GitHub; added the released accession numbers for the six laboratory-generated RNA-seq libraries under BioProjects PRJDB39904 and PRJDB42759; and updated the repository documentation to match the manuscript.
- Standardised supplementary numbering throughout the manuscript, response letter and repository documentation as Figs S1–S5 and Tables S1–S14.
- Expanded the reporting of RNA-seq design, biological replicates, display transformations, statistical tests, exact P-values and computational analysis units.
- Combined each supplementary figure with its legend in one supplementary PDF and used consistent 'designed sequence/HSE-disrupted' terminology throughout the submission files.

Response to the Handling Editor

© 2026. Published by The Company of Biologists under the terms of the Creative Commons Attribution License (https://creativecommons.org/licenses/by/4.0/).

Editor Comment 1 - HSE-disrupted sequence design, motif scanning and methylation control

Editor comment: *Please provide detailed information on how the mutant sequence was generated, how motif scanning was performed, and a control for the DNA methylation analysis.*

Response: We have expanded each of the three requested areas. The revised manuscript provides the native and designed 49-bp sequences, the exact 1-based substitution positions, the TTC-to-GGC and GAA-to-CCG design rules, and the resulting change in GC content. The motif-scanning Methods specify the JASPAR collections, PWM conversion, pseudocount, nucleotide background, strand handling, relative-score calculation, position-level and motif-model summaries, threshold series, and non-redundant interval-merging procedure. For the methylation analysis, we added 1,942 ordinary background transposable elements from the same public methylome and limited the interpretation to basal, unstressed chromatin context. These revisions provide the information needed to reproduce the sequence and motif analyses and add an appropriate genomic control for the methylation comparison.

Changes made: Results; Materials and Methods; Fig. 4; Figs S1-S3; Tables S4-S9.

Editor Comment 2 - Public scripts and sequencing data

Editor comment: *Scripts and sequencing datasets should be deposited.*

Response: The complete analysis workflow is publicly available at https://github.com/BaibhavBarbaruah/ONSEN-Heat-Shock-Element. The repository includes the configuration files, shared functions, package requirements, input provenance, reproducibility matrix, validation procedures and deposited supplementary outputs linked to the final numbering. All six RNA-seq libraries were generated in our laboratory, with three biological replicates per condition, and have been deposited in DDBJ. The three Col-0 non-stressed libraries are available under BioProject PRJDB39904, with accession numbers SAMD01789795, SAMD01789796 and SAMD01789797. The three Col-0 37°C, 24-h heat-stress libraries are available under BioProject PRJDB42759, with accession numbers SAMD01943917, SAMD01943918 and SAMD01943919.

Changes made: Data and resource availability; public GitHub repository and repository documentation.

Editor Comment 3 - Revision files and journal compliance

Editor comment: *Upload identical clean and highlighted Word files, avoid Track Changes, provide separate figure files, and meet display-item and supplementary requirements.*

Response: We prepared text-identical clean and highlighted Word manuscripts without Track Changes or comments. The seven main figures are supplied as separate PDF files. The five supplementary figures and their legends are combined in a single supplementary PDF. The fourteen supplementary tables are supplied as individual Excel files so that the underlying values can be exported and analysed. The manuscript contains seven main display items, within the journal limit of eight. We also completed the journal checklist and added explicit information on RNA-seq biological replicates, computational analysis units, display transformations and statistical procedures.
Changes made: Final submission files; Materials and Methods; Figs 3 and 5 legends; supplementary-information PDF; completed journal checklist.

Response to Reviewer 1

Reviewer 1, Comment 1 - Nature and design of the 12-nt HSE-disrupted sequence

Reviewer comment: *The reviewer requested explicit clarification of whether the mutant was experimental or in silico, why 12 substitutions were introduced, how nucleotides were selected, and which properties were preserved.*

Response: We have clarified this point throughout the manuscript. The sequence is now described as an HSE-disrupted sequence designed in silico, and we state explicitly that it was neither experimentally constructed nor functionally tested. The native 49-bp sequence contains three nTTCn-type units and two nGAAn-type units. Each TTC core was changed to GGC while retaining the terminal C, requiring two substitutions per TTC core; each GAA core was changed to CCG, requiring

© 2026. Published by The Company of Biologists under the terms of the Creative Commons Attribution License (https://creativecommons.org/licenses/by/4.0/).

three substitutions per GAA core. This gives 3×2 + 2×3 = 12 substitutions, at positions 12, 13, 17, 18, 19, 23, 24, 28, 29, 30, 36 and 37. Sequence length and all non-targeted positions were retained, but nucleotide composition was not: GC content increased from 30.61% to 51.02%. The manuscript uses this sequence only to examine predicted motif compatibility.
Changes made: Results section ‘The designed HSE-disrupted sequence…’; Materials and Methods section ‘*ONSEN* HSE sequence selection’; Fig. 1 legend; Table S1.

Reviewer 1, Comment 2 - Robustness across alternative HSE-disrupting designs

Reviewer comment: *The reviewer noted that a single engineered mutant cannot establish that HSE disruption generally creates an AP2/ERF-enriched landscape and requested independent systematic/random designs or natural-variation analysis.*

Response: We agree that the original conclusion was too broad. To determine whether the result depended on a single design, we added 5,000 unique constrained random mutants and exhaustively enumerated all 5,120 valid exact-GC designs, including the original design and 5,119 alternatives. None of the 5,120 exact-GC designs contained an HSF-family hit at the primary threshold, supporting robust predicted HSF loss within the imposed design space. By contrast, AP2/ERF hit number was positively correlated with GC content in the variable-GC library (Spearman rho=0.431, $P<2.2\times10^{-16}$), and the AP2/ERF value of 61 for the designed sequence was not unusual among exact-GC alternatives (median 19; plus-one empirical upper-tail P=0.149). We therefore removed the generalised AP2/ERF conclusion and now describe this result as a composition-sensitive, case-specific collateral motif change.

Changes made: Abstract; Results; Discussion; Fig. S1; Table S4; relevant figure legends and conclusion.
Reviewer 1, Comment 3 - Redundancy among HSF-family PWMs

Reviewer comment: *The reviewer requested clarification that similar HSF PWMs can match the same genomic location and asked for non-redundant sequence-location counts.*

Response: We now report motif-model/position hits separately from non-redundant HSF-compatible sequence locations. Reverse-strand hits were converted to forward-sequence coordinates, and overlapping intervals were then merged across PWM models and strands. In the native 49-bp sequence, 31 raw HSF motif-model/position hits corresponded to one non-redundant compatible location; the designed sequence contained none. Across the sixteen Col-0 terminal candidate windows, 48–75 raw hits corresponded to 7–10 non-redundant locations per window, with a median of eight. We also state clearly that neither measure provides evidence of HSF occupancy *in vivo*.

Changes made: Results; Materials and Methods; Fig. S2; Table S6; repository README.

Reviewer 1, Comment 4 - PWM thresholds and continuous-score analysis

Reviewer comment: *The reviewer requested justification of 0.85 and 0.90, assessment across thresholds, and continuous motif-score distributions to reduce cutoff dependence.*

Response: We agree that the result should not depend on a single motif-score cutoff. The revised Methods define 0.85 as the primary operational high-confidence threshold and 0.90 as a more stringent threshold. We repeated the *ONSEN*-versus-background comparison at 0.80, 0.85, 0.90 and 0.95, and the enrichment was maintained at every threshold (Cliff’s delta=0.991–0.994). We also added three continuous regional summaries: the maximum HSF relative score, the mean of the five highest HSF scores and the median of the ten per-model maximum scores. All three were significantly higher in *ONSEN* windows than in the strict TE background (BH-adjusted Wilcoxon $P\leq2.79\times10^{-8}$). We used the same deposited PWM-scoring framework for the threshold-based and continuous-score analyses so that the results could be compared directly and reproduced within one workflow.

Changes made: Results; Materials and Methods; Fig. S3; Table S8.

© 2026. Published by The Company of Biologists under the terms of the Creative Commons Attribution License (https://creativecommons.org/licenses/by/4.0/).

Reviewer 1, Comment 5 - Interpretation within the Results section

Reviewer comment: *The reviewer suggested moving a literature-comparison section from Results to Discussion.*

Response: We moved the literature-based synthesis from the Results to the Discussion, as suggested. The Results now present the computational observations and statistical comparisons directly. Their relationship to previous *ONSEN*, chromatin and accession studies, together with the limitations of the analysis, is discussed separately.
Changes made: Reorganised Results and Discussion sections.

Reviewer 1, Comment 6 - Figure 7 motif logos and quantitative similarity

Reviewer comment: *The reviewer requested motif logos and a quantitative measure of sequence/motif conservation.*

Response: We added both requested elements. Figure 7C now reports percentage identity and the number of substitutions relative to the 49-bp Col-0 reference. The displayed non-Col-0 variants contain one to four substitutions, corresponding to 91.8–98.0% identity. Figure 7D presents representative JASPAR logos for HSFC1 MA1667.2 (upper) and DOF1.8 MA0981.2 (lower). The Methods and figure legend clarify that these are PWM information-content displays, that the HSFC1 logo is distinct from the MA1667.3 model used in the primary JASPAR 2026 HSF-focused analysis, and that the logos do not provide evidence of transcription-factor binding or occupancy *in vivo*.

Changes made: Fig. 7C,D; Results; Materials and Methods; Fig. 7 legend; Tables S12-S13.

Reviewer 1, Comment 7 - Overstated conclusion

Reviewer comment: *The reviewer stated that the AP2/ERF conclusion was conditional on one designed sequence and could not be generalised.*

Response: We agree that the previous conclusion was too broad and have removed it. The revised manuscript now separates two results: predicted HSF loss was robust across the constrained exact-GC design space, whereas the increase in AP2/ERF compatibility was affected by nucleotide composition and was not unusual among exact-GC alternatives. This distinction is maintained in the Abstract, Results, Discussion, conclusion and the legends for Figs 1, 2 and S1. The designed sequence is therefore presented as a case study rather than as evidence for a general consequence of HSE disruption.

Changes made: Abstract; Results; Discussion; conclusion; Figs 1, 2 and S1; Table S4.

Reviewer 1, Comment 8 - Availability of custom scripts

Reviewer comment: *The reviewer asked whether the custom R scripts would be publicly available.*

Response: Yes. The public repository contains the ordered analysis workflow, shared functions, configuration files, software and package requirements, input provenance, reproducibility matrix, validation script and deposited supplementary outputs. The README explains the PWM-scoring definition, coordinate handling, output mapping and limits of interpretation, allowing the analyses to be reproduced and checked.
Changes made: Data and resource availability; public GitHub repository and README.

Reviewer 1, Comment 9 - RNA-seq accession numbers

Reviewer comment: *The reviewer noted that accession numbers were absent.*

Response: All six RNA-seq libraries were generated in our laboratory, with three biological replicates per condition, and have been deposited in DDBJ. The three Col-0 non-stressed libraries are available under BioProject PRJDB39904, with accession numbers SAMD01789795, SAMD01789796 and SAMD01789797. The three Col-0 37°C, 24-h heat-stress libraries are available under BioProject PRJDB42759, with accession numbers SAMD01943917, SAMD01943918 and SAMD01943919.

Changes made: Data and resource availability.

© 2026. Published by The Company of Biologists under the terms of the Creative Commons Attribution License (https://creativecommons.org/licenses/by/4.0/).

Response to Reviewer 2

Reviewer 2, Comment 1 - Detailed criteria for the 12-nt sequence

Reviewer comment: *A detailed description of the criteria used to generate the 12-nt mutant was missing.*

Response: The revised manuscript now provides the sequence-design rule, all twelve substitution positions, the calculation giving twelve substitutions, the properties retained in the design and the change in nucleotide composition. It also states clearly that the sequence was generated and analysed only in silico. The added constrained-mutant and exhaustive exact-GC analyses test how strongly the predicted motif changes depend on this particular design.
Changes made: Results; Materials and Methods sections '*ONSEN* HSE sequence selection' and 'Constrained-mutant sensitivity analysis'; Fig. S1; Table S4.

Reviewer 2, Comment 2 - Sixteen terminal windows from eight Col-0 loci

Reviewer comment: *The reviewer asked how many ONSEN elements exist in Col-0 and why these elements were selected.*

Response: The analysis set was based on eight full-length *ONSEN* loci historically described in Col-0. We examined fixed 800-bp windows at both ends of these loci, giving sixteen terminal candidate windows that were used consistently for the motif, methylation and RNA-seq analyses. To avoid implying more structural certainty than is supported by the source annotation, we no longer describe these regions as experimentally confirmed exact LTR boundaries or as updated copy-integrity calls. The revised manuscript also distinguishes this historically curated eight-locus set from later refinements of *ONSEN* copy annotation.

Changes made: Results; Materials and Methods; Fig. 3 legend; Discussion and limitations.

Reviewer 2, Comment 3 - Heat-associated methylation change and transcription

Reviewer comment: *The reviewer asked whether heat-associated transcription correlates with DNA methylation change and noted that a heat methylome was absent.*

Response: A methylation–expression correlation cannot be tested with the available datasets. The public methylome was generated from unstressed Col-0 leaves, whereas our RNA-seq libraries were generated from non-stressed and heat-treated whole seedlings; the datasets are neither sample-matched nor tissue-matched. We therefore removed statements implying that heat-induced methylation changes explain the RNA-seq response and now use the methylome only to describe basal chromatin context. We also added a broad ordinary-TE control. CG and CHG methylation did not differ significantly from the background, whereas CHH methylation was higher in *ONSEN* terminal candidate windows (median 53.6% versus 15.8%; BH-adjusted $P=2.06\times10^{-7}$). Published heat-methylation studies are discussed only as independent biological context and not as a substitute for a matched correlation analysis.

Changes made: Abstract; Results; Discussion; Materials and Methods; Fig. 4; Table S9.

Reviewer 2, Comment 4 - Accession-specific heat RNA-seq

Reviewer comment: *The reviewer asked whether RNA-seq data were available to show ONSEN heat responses across the accessions.*

Response: We did not generate accession-specific heat RNA-seq data. We therefore limited the Results to the seed-detected sequence variants and predicted motif patterns supported by the present analysis. Published accession-specific evidence is summarised separately in the Discussion and Table S14, including the Col-0, Ler-1 and Cvi-0 heat-response study of Xu et al. (2024). These datasets are used as independent context, not as functional validation of our motif predictions or as data that can be combined quantitatively with the present analysis.

Changes made: Results; Discussion; Data and resource availability; Table S14.

© 2026. Published by The Company of Biologists under the terms of the Creative Commons Attribution License (https://creativecommons.org/licenses/by/4.0/).

Reviewer 2, Comment 5 - Mechanistic necessity and sufficiency

Reviewer comment: *The reviewer considered the original synthesis too mechanistic because no experiment showed that sequence, methylation and natural variation together are necessary for ONSEN regulation.*

Response: We agree that the earlier wording was too mechanistic. The revised manuscript presents four associated observations: HSF-rich predicted terminal-region architecture, elevated basal CHH methylation relative to ordinary TEs, heat-associated RNA-seq signal over *ONSEN* candidate regions and accession-dependent sequence variation. It does not state that these features, either individually or together, are necessary or sufficient for *ONSEN* activation. We removed wording such as 'determines', 'requires', 'demonstrates binding' and 'methylation change explains transcription', and replaced it with terms that reflect the scope of the analyses, including 'predicted compatibility', 'is associated with', 'basal methylation context' and 'candidate region'. We retained the title because 'integrate' refers to the combined analysis of the sequence, methylation and natural-variation dimensions named in the title; the Abstract, Discussion and conclusion do not use the term in a causal sense.

Changes made: Abstract; Summary statement; Results; Discussion; conclusion and all relevant figure legends.

We believe that these revisions address the points raised by the editor and reviewers. The revised manuscript is accompanied by clean and highlighted Word files, separate main-figure files, the supplementary figures and tables, and the completed journal checklist.

Second decision letter

MS ID#: bio.062799R1

MS Title: Heat-responsive ONSEN long terminal repeats integrate heat shock factor motifs, DNA methylation and natural sequence variation in Arabidopsis

Authors: Baibhav R. Barbaruah, Rahmadani P. Airalangga and Hidetaka Ito

I have now reached a decision on the above manuscript.

The reviewer reports are shown at the bottom of this email.

As you will see, the reviewers gave favourable reports, but raised some critical points that will require amendments to your manuscript. I hope that you will be able to carry these out, because we would like to be able to accept your paper.

As an editor, I am satisfied with the substantial changes that have been done during the first round of revisions. Regarding Reviewer 1's comments, I find that the explanations for the 12 engineered substitutions is now clear and the sentence line 84, that "The native-versus-designed comparison was therefore treated as a case-specific illustration" now clearly sets the limitations of this analysis. The reanalysis of the AP2/Erf motif enrichment is also clarified by the 5,000 random mutants and I appreciate that the emphasis on AP2 motifs is now reduced, but I think that the discussion lines 264-267 should be removed. I agree with the third comment of this reviewer that the number of predicted motifs should be reported as the number of non-redundant motifs throughout the manuscript, since it is meaningless and confusing for the reader otherwise.

Regarding Reviewer 2's comments, please provide the methylation context of non-ONSEN LTR regions for an informed comparison, and a supplementary table with the log2FC values of all genes and TEs upon heat stress.

© 2026. Published by The Company of Biologists under the terms of the Creative Commons Attribution License (https://creativecommons.org/licenses/by/4.0/).

At this stage, we also ask you to ensure your manuscript complies with our formatting guidelines – please see our manuscript preparation guidelines for details. Provided you are able to fully address the referees' comments, we are positive about publication of your paper (we accept over 95% of revision submissions) and therefore hope you won't mind any extra work involved in reformatting your manuscript at this point.

Please upload both a 'clean' version of your Word file, along with a highlighted version clearly showing where you have made changes in the revised manuscript. Please avoid using 'Track changes' in Word files as these are lost in PDF conversion.

I should be grateful if you would also provide a point-by-point response detailing how you have dealt with the points raised by the reviewers in the 'Response to Reviewers' box. Please attend to all of the reviewers' comments. If you do not agree with any of their criticisms or suggestions please explain clearly why this is so.

Reviewer 1

Comments for the author

In the revised manuscript, the authors have updated the motif analysis of the in silico-generated mutants by adding a set of randomly generated control sequences, extended the motif analysis of selected ONSEN LTR regions by validating the presence of the HSF motif across different PWM thresholds, and updated the DNA methylation analysis to include additional background sequences. I appreciate the authors' effort to clarify the motif analysis methodology and to incorporate additional mutational backgrounds. However, these additions have not fully resolved my concerns, and I find the analysis, as currently presented, still difficult to follow.
The artificial introduction of 12 substitutions to specifically abolish all HSE core motifs inevitably results in the loss of predicted HSF motifs and the acquisition of other motifs, which appears to be the main conclusion of this analysis. Since the DNA-binding motifs, particularly those of transcription factor, encoded by degenerate sequence codes, it is expected that any randomly generated DNA sequence will produce a number of predicted binding sites, especially low-complexity motifs (futility theorem https://www.nature.com/articles/nrg1315). I therefore do not understand what this analysis adds or what biological question it is intended to answer.
Similarly, the emphasis on AP2/ERF motifs is confusing. The manuscript ultimately concludes that the observed AP2/ERF enrichment is sequence-specific and largely explained by the chosen nucleotide composition rather than HSE disruption. It is unclear why this result is discussed in such detail.
Finally, I question the biological interpretation of the motif analysis. The authors report 289 high-confidence motif hits within a 49-bp sequence. Given the large number of overlapping predicted binding sites (as I pointed in my previous comments), it is unclear what conclusions can realistically be drawn from them.
As noted in my previous comments and acknowledged by the authors (Lines 148-160), the high counts of predicted motif hits largely reflect redundant, overlapping motif predictions. However, despite acknowledging this and providing the exact number of unique motif positions, the authors continue to use low-information motif hit metric throughout the subsequent analyses and figures.
Taken together, these issues affect a substantial portion of the manuscript's analytical content and remain unresolved despite a prior round of revision. For these reasons, I find the concerns raised to be substantial, and in my opinion, the manuscript is not suitable for publication in its current form.

Reviewer 2

Comments for the author

In the revised manuscript, the authors have done an excellent job of clarifying that their conclusions are based on in silico predictions and have appropriately avoided overstating the evidence for regulatory interdependence between cis-regulatory architecture, epigenetic context, and natural sequence variation. Although the regulation of ONSEN by heat shock is not novel and despite the lack of experimental validation of some of their evidence, the analysis presented

© 2026. Published by The Company of Biologists under the terms of the Creative Commons Attribution License (https://creativecommons.org/licenses/by/4.0/).

represents a useful resource for the plant community. I have, however, two concerns that need to be addressed:
* The authors clearly show that ONSEN LTRs are enriched in HSE motifs and have higher CHH methylation compared to the TE-only background (Figures 3 and 4), but would it be possible to directly compare ONSEN LTRs to the LTRs of other annotated retrotransposons? In other words, are these properties of LTR regions, or are they characteristics of ONSEN LTRs?
* In Figure 5, their analysis of the RNA-seq data clearly shows an upregulation of ONSEN TEs upon exposure to heat stress, while for the outliers examined, the expression does not change; however, a volcano plot should be included showing the global effect of heat shock on TE (and gene) expression. Additionally, a supplementary table with the log2 fold change and adjusted value should be provided, which includes all TEs and genes.
Note: I was able to access the revised text, figures, and supplementary tables but not the supplementary figures

Reviewer's Responses to Questions

Experimental quality
Are experiments performed using appropriate methods that will answer the question (or test the hypothesis or support the observations) posed by the authors? Is the right tool used for the job?
If 'No', please indicate reasons in Comments for Author box below.
Reviewer #1:
- Yes

Reviewer #2:
- Yes

Were the data analyzed using appropriate statistical tests?
If 'No', please indicate reasons in Comments for Author box below.
Reviewer #1:
- Yes

Reviewer #2:
- Yes

Reproducibility
Were experiments performed using adequate number of biological replicates?
If 'No', please indicate reasons in Comments for Author box below.
Reviewer #1:
- Yes

Reviewer #2:
- Yes

Is there sufficient raw data to assess the rigor of the analysis?
If 'No', please indicate reasons in Comments for Author box below.
Reviewer #1:
- Yes

Reviewer #2:
- Yes

Does the methods section provide sufficient detail to permit reproducibility?
If 'No', please indicate reasons in Comments for Author box below.
Reviewer #1:
- Yes

© 2026. Published by The Company of Biologists under the terms of the Creative Commons Attribution License (https://creativecommons.org/licenses/by/4.0/).

Reviewer #2:
- Yes

Completeness
Are the manuscript's conclusions supported by the data?
If 'No', please indicate reasons in Comments for Author box below.
Reviewer #1:
- No

Reviewer #2:
- Yes

Scholarship
Do the authors cite and discuss the merits of data that would argue for and against their conclusion?
If 'No', please indicate reasons in Comments for Author box below.
Reviewer #1:
- Yes

Reviewer #2:
- Yes

Does the manuscript title & abstract accurately reflect the contents of the manuscript, without hyperbole?
If 'No', please indicate reasons in Comments for Author box below.
Reviewer #1:
- No

Reviewer #2:
- Yes

Second revision

Author response to reviewers' comments

Dear Dr Monniaux,

We thank you and the reviewers for carefully assessing the revised manuscript. We have made the remaining changes requested in the decision letter. In particular, we removed the remaining extended AP2/ERF discussion, replaced the redundant motif-hit counts with non-redundant coordinate-based summaries, added a direct comparison with non-*ONSEN* LTR retrotransposons for the methylation analysis, and added genome-wide gene and TE differential-expression results. We also checked the manuscript, figures, legends and supplementary files again to make sure the numbering is consistent throughout.

Overview of the major revisions

- Removed the extended AP2/ERF discussion requested by the editor and deleted the standalone AP2/ERF supplementary table.
- Replaced redundant PWM model-position counts with non-redundant exact-coordinate placements throughout; retained strict overlap-merged HSF regions as a clearly labelled sensitivity metric.

© 2026. Published by The Company of Biologists under the terms of the Creative Commons Attribution License (https://creativecommons.org/licenses/by/4.0/).

- Updated the native 49-bp and sixteen Col-0 terminal-window results, all genome-wide comparisons, methods, legends and supplementary descriptions to the same coordinate-based definition.
- Added a direct element/copy-level methylation comparison between *ONSEN* termini and 779 annotation-defined non-*ONSEN* LTR retrotransposons, with window-level and broad-TE sensitivity analyses.
- Added gene and TE volcano plots to Fig. 5 and a complete supplementary workbook containing log2 fold changes and BH-adjusted P-values for all 32,833 genes and 31,189 TEs.
- Renumbered the final 13 supplementary tables in manuscript citation order after deletion of the AP2/ERF table; genome-wide differential expression is Table S9, candidate-window RNA-seq is Table S10 and accession analyses are Tables S11–S13. The final supplementary-figure set is Figs S1–S4.
- Prepared text-identical clean and highlighted Word manuscripts without Track Changes and separated the seven main figures from the combined supplementary-figure PDF.

Response to the Handling Editor

Editor Comment 1 - AP2/ERF discussion and non-redundant motif reporting

Editor comment: 'The reanalysis of the AP2/Erf motif enrichment is also clarified by the 5,000 random mutants and I appreciate that the emphasis on AP2 motifs is now reduced, but I think that the discussion lines 264-267 should be removed. I agree with the third comment of this reviewer that the number of predicted motifs should be reported as the number of non-redundant motifs throughout the manuscript, since it is meaningless and confusing for the reader otherwise.'

Response: We agree. We removed the extended AP2/ERF discussion and deleted the standalone AP2/ERF supplementary table. Figure 2B is kept only to show the PWM-score changes for this particular engineered sequence. We now state clearly that changes outside the HSF family are specific to this sequence and are not used for a general biological conclusion. We also changed the main motif-count measure to non-redundant exact-coordinate placements, so predictions with the same 1-based start and end coordinates are counted once across PWM models and strands. Using this definition, the native 49-bp sequence has 20 HSF placements and the designed sequence has none, while the sixteen Col-0 terminal windows have 33–49 placements each. As an additional check, the stricter overlap-merging analysis gives 7–10 broader HSF-compatible regions per window. We used the same definitions and values throughout the Abstract, Results, Discussion, Methods, legends and Tables S1 and S3–S7.

Changes made: Abstract; Introduction; Results; Discussion; Materials and Methods; Figs 1, 3, S1 and S2; Tables S1 and S3–S7; supplementary numbering.

Editor Comment 2 - Direct non-ONSEN LTR methylation context

Editor comment: 'Regarding Reviewer 2's comments, please provide the methylation context of non-ONSEN LTR regions for an informed comparison, and a supplementary table with the log2FC values of all genes and TEs upon heat stress.'

Response: We added the requested direct comparison. For the main analysis, annotation-defined non-*ONSEN* LTR retrotransposons were represented by fixed terminal regions and the left and right termini were combined at the element level, so the two termini of the same element were not treated as independent samples. This gave 779 non-*ONSEN* LTR-retrotransposon elements. CG and CHG methylation were not significantly different from *ONSEN* (BH-adjusted P=0.849 for both), whereas CHH methylation was higher at *ONSEN* termini (median 47.09% versus 20.06%; BH-adjusted P=0.00864; Cliff's δ=0.612). The window-level and broad ordinary-TE analyses showed the same CHH pattern. Figure 4A and Table S8 now use this direct LTR-retrotransposon comparison as the main methylation comparison.

Changes made: Results; Discussion; Materials and Methods; Fig. 4; Table S8; Fig. 4 legend.

Editor Comment 3 - Genome-wide gene and TE differential expression

Editor comment: 'Regarding Reviewer 2's comments, please provide the methylation context of non-ONSEN LTR regions for an informed comparison, and a supplementary table with the log2FC values of all genes and TEs upon heat stress.'

Response: We added genome-wide differential-expression analyses and kept all annotated features in Table S9, not only the significant ones. The table contains all 32,833 genes and 31,189

© 2026. Published by The Company of Biologists under the terms of the Creative Commons Attribution License (https://creativecommons.org/licenses/by/4.0/).

transposable elements, with log2 fold changes, raw and BH-adjusted P-values where available, filtering status and differential-expression calls. DESeq2 returned model estimates for 25,912 genes, raw P-values for 25,911 and non-missing BH-adjusted P-values for 23,922 after independent filtering; the other rows are still included with the appropriate missing entries. For TEs, 2,101 loci passed expression filtering and were tested. Using BH-adjusted $P<0.05$ and $|\log2 \text{ fold change}|\geq 1$, 3,912 genes and 692 TEs were upregulated, whereas 4,185 genes and 397 TEs were downregulated. The new global gene and TE volcano plots are shown in Fig. 5D,E. All eight canonical *ONSEN* loci are labelled in Fig. 5E and were classified as upregulated. We have kept the multimapping caveat for repetitive sequences in the manuscript.
Changes made: Results; Materials and Methods; Fig. 5D,E; Table S9; Fig. 5 legend; Data and resource availability.

Editor Comment 4 - Revision-file compliance
Editor instructions: 'Please upload both a clean version of your Word file, along with a highlighted version clearly showing where you have made changes in the revised manuscript. Please avoid using Track changes in Word files as these are lost in PDF conversion.' The formatting instructions additionally request a separate file for each main figure and require supplementary items to be uploaded separately.
Response: We prepared clean and highlighted Word manuscripts with identical text and without Track Changes or comments. The seven main figures are supplied as separate PDF files. Figs S1–S4 are combined in one supplementary-figure PDF, with each complete legend directly below its figure on the same page. After removing the former AP2/ERF Table S3, the final set contains 13 supplementary Excel tables. We checked the table titles, worksheet names, manuscript citations and figure numbering against the final files.
Changes made: Final submission file set and supplementary numbering.

Response to Reviewer 1

Reviewer 1, Comment 1 - Biological value of the engineered sequence
Reviewer comment: 'The artificial introduction of 12 substitutions to specifically abolish all HSE core motifs inevitably results in the loss of predicted HSF motifs and the acquisition of other motifs, which appears to be the main conclusion of this analysis. Since the DNA-binding motifs, particularly those of transcription factor, encoded by degenerate sequence codes, it is expected that any randomly generated DNA sequence will produce a number of predicted binding sites, especially low-complexity motifs (futility theorem https://www.nature.com/articles/nrg1315). I therefore do not understand what this analysis adds or what biological question it is intended to answer.'
Response: We agree that the purpose of this analysis needed to be narrower. We now present it only as a case-specific HSF-focused computational control, not as a general model of HSE evolution or motif creation. The selected design and all 5,119 exact-GC alternatives have zero non-redundant HSF motif-coordinate placements at the primary threshold. We also state clearly that this is a computational result and does not demonstrate binding or regulatory function in vivo. We therefore do not use the collateral changes in other TF families to make a general biological conclusion. The extended AP2/ERF interpretation and the dedicated AP2/ERF supplementary table have been removed.
Changes made: Abstract; Results subsection title and text; Discussion; Table S3; Fig. 2 legend.

Reviewer 1, Comment 2 - Excessive AP2/ERF emphasis
Reviewer comment: 'Similarly, the emphasis on AP2/ERF motifs is confusing. The manuscript ultimately concludes that the observed AP2/ERF enrichment is sequence-specific and largely explained by the chosen nucleotide composition rather than HSE disruption. It is unclear why this result is discussed in such detail.'
Response: We agree. We removed the extended AP2/ERF discussion and the AP2/ERF-focused supplementary figure and standalone table. Figure 2B is retained simply to show the scores obtained for this particular engineered sequence. The text now makes clear that the changes outside the HSF family are sequence-specific collateral effects and are not used for a general biological conclusion.
Changes made: Abstract; Introduction; Results; Discussion; supplementary Fig. S1 removed and remaining figures renumbered; former Table S3 removed; Fig. 2 legend.

© 2026. Published by The Company of Biologists under the terms of the Creative Commons Attribution License (https://creativecommons.org/licenses/by/4.0/).

Reviewer 1, Comment 3 - Redundant motif counts

Reviewer comment: 'Finally, I question the biological interpretation of the motif analysis. The authors report 289 high-confidence motif hits within a 49-bp sequence. Given the large number of overlapping predicted binding sites (as I pointed in my previous comments), it is unclear what conclusions can realistically be drawn from them. As noted in my previous comments and acknowledged by the authors (Lines 148-160), the high counts of predicted motif hits largely reflect redundant, overlapping motif predictions. However, despite acknowledging this and providing the exact number of unique motif positions, the authors continue to use low-information motif hit metric throughout the subsequent analyses and figures.'

Response: We agree. We no longer report the 289 overlapping motif-model/position predictions as a motif count. The main count used in the revised manuscript is the number of non-redundant exact-coordinate placements. Distinct PWM model identities are reported only when the analysis is specifically about model composition. Predictions from different models or strands that have the same coordinates are counted once. As a stricter check, overlapping HSF intervals are also merged transitively. With these definitions, the native 49-bp sequence has 20 exact-coordinate HSF placements (one strict overlap-merged region) and the designed sequence has none. The sixteen Col-0 terminal windows have 33–49 exact-coordinate placements and 7–10 strict overlap-merged regions each. We have used these terms consistently in the figures, tables, Methods and legends.

Changes made: Abstract; Results; Discussion; Materials and Methods; Figs 1, 3, 7, S1–S4; Tables S1 and S3–S12; all relevant legends.

Reviewer 1, Comment 4 - Overall interpretation

Reviewer comment: 'Taken together, these issues affect a substantial portion of the manuscript's analytical content and remain unresolved despite a prior round of revision. For these reasons, I find the concerns raised to be substantial, and in my opinion, the manuscript is not suitable for publication in its current form.'

Response: We understand the concern. We therefore revised the manuscript so that the main motif result is the non-redundant HSF-coordinate enrichment of *ONSEN* terminal regions compared with the harmonised 1,930-region non-*ONSEN* TE background. The same enrichment is supported across four thresholds and the continuous-score analyses. The engineered-sequence analysis is now kept only as a case-specific computational control. We also added the direct LTR-retrotransposon methylation comparison and the global gene/TE expression analyses as additional context, but we do not use them to claim causality. We believe these changes address the reviewer's concern and make clearer what each analysis shows and what it does not.

Changes made: Entire manuscript narrative and linked supplementary package.

Response to Reviewer 2

Reviewer 2, Comment 1 - Direct LTR-retrotransposon comparison

Reviewer comment: 'The authors clearly show that ONSEN LTRs are enriched in HSE motifs and have higher CHH methylation compared to the TE-only background (Figures 3 and 4), but would it be possible to directly compare ONSEN LTRs to the LTRs of other annotated retrotransposons? In other words, are these properties of LTR regions, or are they characteristics of ONSEN LTRs?'

Response: We added the direct comparison requested by the reviewer. The main methylation analysis now compares *ONSEN* with 779 annotation-defined non-*ONSEN* LTR retrotransposons at the element/copy level, so paired termini from the same element are not treated as independent samples. CG and CHG methylation were not significantly different, whereas CHH methylation was higher at *ONSEN* (47.09% versus 20.06%; adjusted P=0.00864). Figure 4 and Table S8 now separate this direct LTR comparison from the broader ordinary-TE and selected-outlier analyses.

Changes made: Results; Discussion; Materials and Methods; Fig. 4; Table S8; Fig. 4 legend.

Reviewer 2, Comment 2 - Global gene and TE volcano plots and full results

Reviewer comment: 'In Figure 5, their analysis of the RNA-seq data clearly shows an upregulation of ONSEN TEs upon exposure to heat stress, while for the outliers examined, the expression does not change; however, a volcano plot should be included showing the global effect of heat shock on TE (and gene) expression. Additionally, a supplementary table with the log2 fold change and adjusted value should be provided, which includes all TEs and genes.'

Response: We added separate gene and TE volcano plots as Fig. 5D,E and provide the complete results in Table S9. The workbook includes all annotated features rather than only the significant

© 2026. Published by The Company of Biologists under the terms of the Creative Commons Attribution License (https://creativecommons.org/licenses/by/4.0/).

ones. It gives feature identifiers, expression summaries, model log2 fold changes, standard errors or statistics where applicable, raw and BH-adjusted P-values, filtering/testing status and differential-expression calls. For genes, we distinguish rows with missing adjusted P-values after DESeq2 independent filtering from rows without model estimates. We also included a separate sheet with the eight canonical *ONSEN* loci so they can be checked directly against the global TE results.
Changes made: Results; Materials and Methods; Fig. 5D,E; Table S9; Fig. 5 legend; Data and resource availability.

Reviewer 2, Comment 3 - Supplementary-figure accessibility
Reviewer note: 'I was able to access the revised text, figures, and supplementary tables but not the supplementary figures.'
Response: We combined the final four supplementary figures into a single supplementary-figure PDF and placed each complete legend directly below the corresponding figure. We checked all four pages after making the file. Figure S4C was also checked against the accession summary. The manuscript now cites only Figs S1–S4, matching the supplied PDF.
Changes made: Supplementary-figure PDF and all supplementary-figure citations and legends.
We believe that these revisions address the remaining points raised in the second-round decision. We have checked the clean and highlighted manuscripts, response letter, seven main-figure PDFs, supplementary-figure PDF and Tables S1–S13 against one another and against the public repository.

Third decision letter

MS ID#: bio.062799R2

MS Title: Heat-responsive ONSEN long terminal repeats integrate heat shock factor motifs, DNA methylation and natural sequence variation in Arabidopsis

Authors: Baibhav R. Barbaruah, Rahmadani P. Airalangga and Hidetaka Ito

I am happy to tell you that your manuscript has been accepted for publication in Biology Open, pending our standard publication integrity checks. It was accepted on 17th August 2026.

© 2026. Published by The Company of Biologists under the terms of the Creative Commons Attribution License (https://creativecommons.org/licenses/by/4.0/).
